# Role of tristability in the robustness of the differentiation mechanism

**DOI:** 10.1371/journal.pone.0316666

**Published:** 2025-03-19

**Authors:** Corentin Robert, Francisco Prista von Bonhorst, Geneviève Dupont, Didier Gonze, Yannick De Decker

**Affiliations:** 1 Nonlinear Physical Chemistry Unit, Université Libre de Bruxelles (ULB), Brussels, Belgium; 2 Unit of Theoretical Chronobiology, Université Libre de Bruxelles (ULB), Brussels, Belgium; Utrecht University, NETHERLANDS, KINGDOM OF THE

## Abstract

During cell differentiation, identical pluripotent cells undergo a specification process marked by changes in the expression of key genes, regulated by transcription factors that can inhibit the transcription of a competing gene or activate their own transcription. This specification is orchestrated by gene regulatory networks (GRNs), encompassing transcription factors, biochemical reactions, and signalling cascades. Mathematical models for these GRNs have been proposed in various contexts, to replicate observed robustness in differentiation properties. This includes reproducible proportions of differentiated cells with respect to parametric or stochastic noise and the avoidance of transitions between differentiated states. Understanding the GRN components controlling these features is crucial. Our study thoroughly explored an extended version of the Toggle Switch model with auto-activation loops. This model represents cells evolving from common progenitors in one out of two fates (***A*** or ***B***, bistable regime) or, additionally, remaining in their progenitor state (***C***, tristable regime). Such a differentiation into populations with three distinct cell fates is observed during blastocyst formation in mammals, where inner cell mass cells can remain in that state or differentiate into epiblast cells or primitive endoderm. Systematic analysis revealed that the existence of a stable non-differentiated state significantly impacts the GRN’s robustness against parametric variations and stochastic noise. This state reduces the sensitivity of cell populations to parameters controlling key gene expression asymmetry and prevents cells from making transitions after acquiring a new identity. Stochastic noise enhances robustness by decreasing sensitivity to initial expression levels and helping the system escape from the non-differentiated state to differentiated cell fates, making the differentiation more efficient.

## Introduction

During development, cells continuously divide and differentiate to form the various organs of a living being. The ability of a multipotent cell to choose a particular cell fate often involves a change in the expression of a key gene, typically encoding transcription factors linked to the differentiation process [1, 2]. The expression of these key genes within a cell is regulated by the presence or absence of associated transcription factors, that can either inhibit the transcription of a competing gene or activate the transcription of their own gene [1–3]. The differentiation of multipotent cells is regulated by numerous interactions between transcription factors, biochemical reactions, signalling cascades, etc. These processes are collectively referred to as a gene regulatory network (GRN).

**Fig 1:**
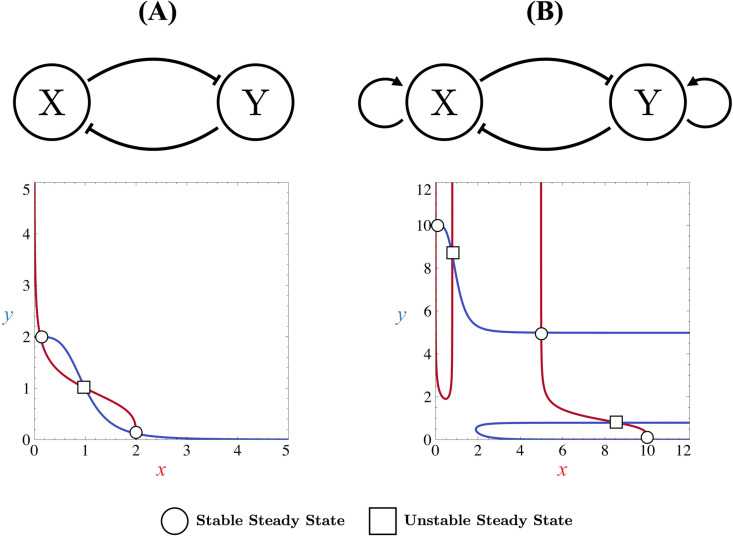
Topologies of simple GRNs governing cell differentiation mechanism. Schematic representations of two GRNs: (A) a two-component mutually inhibitory feedback loop without autoactivation (Toggle Switch) and (B) the same loop with autoactivations (Self-activating Toggle Switch). In these circuits,  ⊣  arrows indicate gene inhibition in the transcription process, while  →  arrows represent gene activation in the transcription process. For each topology, the corresponding phase portrait is shown. Stable and unstable steady states are marked with circles and squares, respectively. Nullclines are included in the phase portraits, with the *x*-nullcline shown in red and the *y*-nuclline in blue.

Numerous theoretical models [4] have been proposed to comprehend these mechanisms and to grasp how such networks drive cell specification and differentiation. One of the earliest models that describe how two key transcription factors can mutually inhibit each other, thus explaining how cells differentiate, is the well-known Toggle Switch proposed by Gardner et al. [5] (Fig 1A). This model can display bistability, a crucial behaviour for cell differentiation [1,2,5,6] where a progenitor cell can evolve in one out of two different differentiated states, each characterized by the exclusive expression of one key transcription factor, marking the cell’s commitment to a specific lineage. In contrast, a non-differentiated state represents a state where both transcription factors are coexpressed at similar levels, indicating a multipotent or uncommitted status. However, while sufficient for differentiation, the Toggle Switch only represents systems where a cell exists in one of two differentiated states, with one transcription factor being expressed while the other is not (bistable case), or in a single non-differentiated state where both transcription factors are coexpressed (monostable case). The coexistence of differentiated and non-differentiated cells is therefore not possible in such models.

Nevertheless, certain developmental systems may exhibit this coexistence [1–3,7–11]. This happens, for instance, during cell specification in pre-implantation mammalian embryogenesis where ICM cells give rise to Epiblast (Epi, high level of Nanog transcription factor), and Primitive Endoderm (PrE, high level of Gata6 transcription factor) cells or maintain their pluripotency [9,10,12,13]. This preservation of an uncommitted state has also been observed during the cell specification of the common myeloid progenitor into erythroid cells (high level of Gata1 transcription factor) or myeloid cells (high level of PU.1 transcription factor) [1–3,14]. In general, a common characteristic of systems that preserve a non-differentiated state during and after the differentiation process is the presence of autoactivations of the transcription factors that govern the differentiation mechanism [2].

From a modelling perspective, it has been demonstrated that introducing the ability for transcription factors to activate their own expression allows for the presence of a stable, non-differentiated state that coexists with differentiated stable states [1–3,7,11,15], which is consistent with experimental observations. Such a regime is called, in the context of dynamical systems, tristability. Several models have thus been developed in this direction [4], and one of them will be the subject of our analysis in this work (Fig 1B).

Studying simple models offers insights into how multipotent cells differentiate reproducibly (across various cell populations despite environmental fluctuations) and heterogeneously (resulting in distinct states despite initial cellular similarities). These models aim to explain the robustness that characterizes the differentiation process, which remains highly regulated to face environmental perturbations. Notably, prior to differentiation, the symmetry of the GRNs is essential to maintain pluripotency. Differentiation, however, requires a symmetry-breaking event, which can be triggered by intrinsic noise (stochastic fluctuations in transcription factor concentrations) or extrinsic noise (variations in kinetic parameters related to gene transcription processes). It is, therefore, crucial to understand which components of the GRN confer robustness to the differentiation mechanism despite perturbations and asymmetries in order to elucidate the mechanisms underlying differentiation. In this work, we model environmental perturbations through stochastic dynamics of transcription factors and asymmetries through variations in transcriptional kinetic parameters. To understand which components of the GRNs govern these robustness features and to study the role of tristability rather than bistability, we here focus on a minimal 2-variable system.

Current research on decision-making processes has primarily explored configurations to achieve tristability [2–4,16] and examined how network topology affects the robustness of this regime [11] against noise. By analysing bifurcation diagrams and potential landscapes under different parametric configurations, these studies emphasize that networks featuring mutual inhibition between two key genes, coupled with self-activation, are good candidates for robust architectures. This robustness against parametric perturbations is attributed to the stabilization of the non-differentiated steady state (i.e., tristability). These findings have also been supported by statistical methods [16–18] developed to quantify the robustness of a regulatory networks against randomly imposed parametric perturbations.

While these studies provide valuable insights for designing robust cellular differentiation models by proposing suitable topologies, they do not address how tristability and gene self-activation influence the dynamics of these systems or uncover the origin of this robustness from a dynamical systems theory perspective. Moreover, they have not systematically investigated how parametric asymmetries affect the system dynamics nor scanned the full parameter space to identify specific regimes. They lack detailed analyses of phase space structures and trajectories, which are essential for understanding the dynamical mechanisms underpinning robustness.

Our work addresses this gap by providing a comprehensive analysis that combines statistical approaches with a detailed examination of the system’s dynamical properties. Specifically, we investigate how tristability specifically contributes to the robustness of different key properties of the differentiation process in the context of a two-genes GRN against perturbations and network-level asymmetries. To this aim, we first reparameterized Huang’s model [3] to distinguish between parameters controlling stability regimes and those affecting the symmetry of the GRN, enabling a focused study of parametric perturbations and their effects on the dynamic and stationary behaviours of the system. This reparameterization enables us to specifically investigate the role of symmetry breaking and allows us to explore its influence on the phase portrait and the robustness of key features associated with the differentiation process with greater precision—an aspect not systematically addressed in prior studies.

Additionally, through an exhaustive screening of the parameter space, we provide now a complete description of Huang’s model, analysing the effects of parameters and asymmetries on stability domains within one- and two-parameter bifurcation diagrams. This approach uncovers particular regimes, such as conditions under which tristability is achievable, the emergence of ghost states, and the overall dynamical richness of this model. For each parametric domain, our work addresses both the phase space structures and trajectories in perturbed and unperturbed cases and the effect of these perturbations on key properties of the differentiation process. Together, these two perspectives provide insights into why tristable configurations decrease the sensitivity of key differentiation properties to noise or parametric perturbations based on the intrinsic properties of the system.

## Models

The GRN that we will study in this work consists of a 2-component motif where two proteins (transcription factors) can have an impact on the transcription process of their own gene or of another competitive gene. More specifically, in a cellular differentiation context, transcription factors will repress the transcription process of the competitive gene while they will activate the transcription process of their own gene as schematized in Fig 1B. No distinctions are made between mRNA and protein species. The variables that will be considered are the concentration of the proteins.

In the absence of autoactivations, the GRN in Fig 1B reduces to a Toggle Switch [5]. As mentioned in the introduction, while the Toggle Switch is sufficient for representing the differentiation process, it exhibits only bistability, failing to maintain the non-differentiated state as a stable steady state. We define autoactivation as the ability of transcription factors to activate their own transcription, as represented in Fig 1B. Models incorporating autoactivation typically predict the coexistence of more than two stable steady states. One such model was proposed by Huang [3] where the dynamics of concentrations *x* and *y* associated with the X and Y transcription factors in a single isolated cell are governed by the following differential equations:


$$\frac{dx}{dt}=v_{x}\frac{K_{ix}{}^{n}}{K_{ix}{}^{n}+y^{n}}+w_{x}\frac{x^{n}}{K_{ax}{}^{n}+x^{n}}-k_{x}x$$ (1)

$$\frac{dy}{dt}=v_{y}\frac{K_{iy}{}^{n}}{K_{iy}{}^{n}+x^{n}}+w_{y}\frac{y^{n}}{K_{ay}{}^{n}+y^{n}}-k_{y}y$$ (2)

The first term in each right-hand side corresponds to the inhibition of transcription of one gene by the other and is modelleusing a Hill function. The parameter $v_{j}$ (with j=x or *y*) corresponds to the maximum rate of protein *j* production in the cell, *n* is the Hill coefficient associated with transcription factors X and and could represent the number of transcription factor binding sites on the gene coding for the protein, $K_{ij}$ is an equilibrium constant that indicates how much the transcription factor is bound to the transcription site. The second term represents the activation of gene transcription by its own corresponding protein. Like the inhibition term, the autoactivation term is represented by a Hill function. The parameter $w_{j}$ is the maximum rate of autoactivation of protein *j* and $K_{aj}$ is an equilibrium constant that scales how much the transcription factor is bound to the transcription site linked to the autoactivation process. The last terms correspond to a degradation of proteins that follows first-order kinetics. In this work, we will assume that all Hill coefficients are equal to 4. This value is selected to introduce sufficient nonlinearity for the system to generate bistability and tristability. Additionally, the Hill coefficients for the inhibition and autoactivation terms are kept similar, ensuring that both Hill functions have a comparable influence on the system’s dynamics.

This implementation of the additive autoactivation terms is premised on the assumption that gene transcription’s autoactivation operates independently from inhibition. Additional key assumptions are made within this framework: First, transcription dynamics adhere to Hill-type kinetics, implying cooperativity among activator binding sites and repressor binding sites (similar to activator and inhibitor complex formation). Second, there is no competition among various transcription factors for the same binding site. Finally, the presence of an activator at one site does not influence the repressor’s binding rate at another site, and vice versa. Given the independence between inhibition and autoactivation, this logic is commonly referred to as the « OR » logic [10,19], meaning that each term can individually influence the system’s dynamic. It is the reason why, in the limit where transcription factors do not autoactivate themselves, this model reduces to the Toggle Switch.

The validity of this hypothesis of independence between inhibition and autoactivation [4] agrees with experimental data [2–4,7,14] within the framework of the GATA1/PU.1 system, which are the key transcription factors governing myeloid progenitor cell differentiation. Furthermore, the appeal of this hypothesis lies in its ability to model the system without requiring precise knowledge of transcription factor interdependencies. This model offers then a broad initial approach to this type of GRN (cross-inhibition and activation).

To study the robustness of the GRN against parametric deviations from parametric symmetry ($v_{x}=v_{y}$), ($w_{x}=w_{y}$),…, we introduce new parameters *Δ*. These parameters take into account the extent to which the system can cope with such asymmetries, starting from a symmetric GRN, before completely (de)favouring the transcription of one of the two genes. These parameters can therefore be interpreted as order parameters since they have an impact on the symmetry of the system and introduce a symmetry breaking. We can then apply the following transformation on every parameter:


$$v_{x}\to v\left(1+\Delta_{FI}\right);v_{y}\to v\left(1-\Delta_{FI}\right)$$ (3)

$$w_{x}\to w\left(1+\Delta_{FA}\right);w_{y}\to w\left(1-\Delta_{FA}\right)$$ (4)

$$K_{ix}^{n}\to K_{i}^{n}{\left(1+\Delta_{KI}\right)}^{n};K_{iy}^{n}\to K_{i}^{n}{\left(1-\Delta_{KI}\right)}^{n}$$ (5)

$$K_{ax}^{n}\to K_{a}^{n}{\left(1+\Delta_{KA}\right)}^{n};K_{ay}^{n}\to K_{a}^{n}{\left(1-\Delta_{KA}\right)}^{n}$$ (6)

$$k_{x}\to k\left(1+\Delta_{D}\right);k_{y}\to k\left(1-\Delta_{D}\right)$$ (7)

where $\Delta_{FI}$, $\Delta_{FA}$, $\Delta_{KI}$, $\Delta_{KA}$, *Δ*_*D*_ are bounded between −1 and 1. Depending on the Δ values, the system is symmetric (*Δ* = 0) or asymmetric (*Δ* ≠ 0.

To simplify Eqs (1) and (2), we can use the following dimensionless variables/parameters: $\tilde{x}=xK_{i}^{-1}$, $\tilde{y}=yK_{i}^{-1}$, $F_{I}=v{\left(K_{i}k\right)}^{-1}$, $F_{A}=w{\left(K_{i}k\right)}^{-1}$, $\kappa =K_{a}K_{i}^{-1}$, $\tilde{t}=kt$ to obtain:


$$\frac{dx}{dt}=F_{I}\left(1+\Delta_{FI}\right)\frac{{\left(1+\Delta_{KI}\right)}^{n}}{{\left(1+\Delta_{KI}\right)}^{n}+y^{n}}+F_{A}\left(1+\Delta_{FA}\right)\frac{x^{n}}{{\left(1+\Delta_{KA}\right)}^{n}\kappa^{n}+x^{n}}-\left(1+\Delta_{D}\right)x$$ (9)

$$\frac{dy}{dt}=F_{I}\left(1-\Delta_{FI}\right)\frac{{\left(1-\Delta_{KI}\right)}^{n}}{{\left(1-\Delta_{KI}\right)}^{n}+x^{n}}+F_{A}\left(1-\Delta_{FA}\right)\frac{y^{n}}{{\left(1-\Delta_{KA}\right)}^{n}\kappa^{n}+y^{n}}-\left(1-\Delta_{D}\right)y$$ (10)

where tildes on the variables *x*, *y* and *t* have been omitted for clarity.

Introducing these new parameters allows us to separate the parameters into two groups. First, we have the *core* parameters $F_{I}$ (the inhibition force), $F_{A}$ (the autoactivation force) and *κ* (the dissociation constants ratio) which characterize the properties (dynamics, multistability, etc.) of a fully symmetric GRN. Second, the asymmetry parameters have an impact on the symmetric behaviour of the GRN. Secondly, we have the *asymmetry* parameters $\Delta_{FI},\Delta_{FA},\Delta_{KA},\Delta_{KI},\Delta_{D}$ which control the (a)symmetry of the GRN and determine if one or another variable, i.e., cellular fate, is favoured.

In this work, we consistently assume that the system starts from a low, symmetric initial condition ($x_{0}=y_{0}=0$), representing a progenitor cell in an early, non-differentiated state. This choice contrasts with the more common approach of initializing the system from an advanced progenitor state (i.e., $\left(x_{0}=y_{0}\right)\neq 0$) [3,20]. Our decision is motivated by several factors. First, in developmental biological systems [21–24], progenitor cells typically exhibit low expression levels of transcription factors during the initial stages of differentiation, aligning with the state we aim to model. Second, starting from a low, symmetric initial condition allows us to highlight the crucial role of noise and early dynamics fluctuations in influencing differentiation pathways, regardless of the stability of the steady state that represent the non-differentiated cell fate. By selecting symmetric initial conditions, we eliminate bias in evaluating the role of inherent asymmetries within the gene regulatory network (GRN) and ensure that observed differentiation results from network dynamics rather than an initial variability. This consistent initial condition choice ($x_{0}=y_{0}=0$) applies to all simulations, ensuring comparability across different parameter regimes. Consequently, the system remains in a non-differentiated state unless driven by either intrinsic stochasticity or asymmetry parameters, aligning with the biological concept of stemness or multipotency, where undifferentiated cells maintain the potential for multiple differentiation outcomes.

Finally, to account for stochasticity in such a system, we used the chemical master equation which is an ordinary differential equation for the probability of having a certain number of molecules of X and Y. In such an equation, the transition probabilities are functions of the extensivity parameter Ω which controls the total number of proteins, and therefore the level of molecular noise in the system. In this work, we will assume that the Hill functions in Eqs (9) and (10) are directly linked to transition probabilities. Considering this approximation, the various production and degradation processes for our model are summarized in the S1 Table. Rather than trying to find an analytical solution to this master equation, we simulated their associated stochastic trajectories as a function of time using the Gillespie algorithm [25, 26]. This allows us to explore the dynamics of the concentrations of both transcription factors in the system and look at the probability distributions $P\left(X,Y\right)$ to have a certain amount of *X* and *Y* molecules if the simulation is repeated a certain number of times with the same initial condition and the same parametric configuration.

## Results

### Phase space and bifurcation analysis

**Fig 2:**
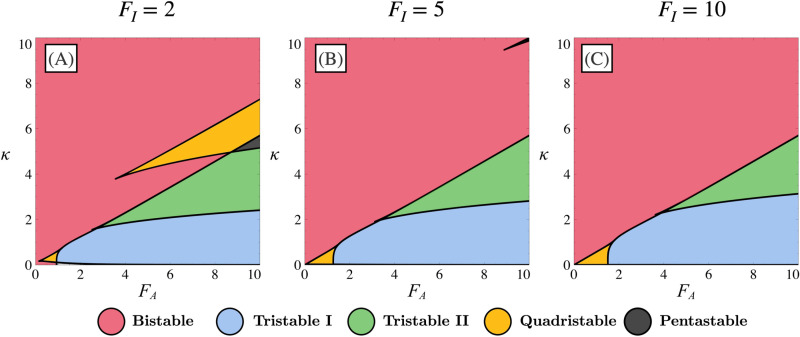
Multistability domains for the GRN. Stability diagrams for the system defined by Eqs (9) and (10) as a function of parameters $F_{A}$ and *κ*. Panels (A), (B), and (C) correspond to different values of inhibition force $F_{I}$ ((A) $F_{I}=2$, (B) $F_{I}=5$, (C) $F_{I}=10$). Each coloured zone represents a distinct stability domain as indicated.

In the case where the system is symmetric, each parameter Δ is equal to 0 and multiple multistability domains can be found depending on the values of $F_{I}$, $F_{A}$ and *κ*. To systematically analyse the different possible regimes, we show in Fig 2 a stability diagram of *κ* as a function of $F_{A}$ for several values of $F_{I}$. Although the system exhibits various regimes, we will focus only on the bistable (red) and tristable (blue and green) regimes, since these are the ones that make sense from a biological perspective.

### Bistability in the symmetric case

**Fig 3:**
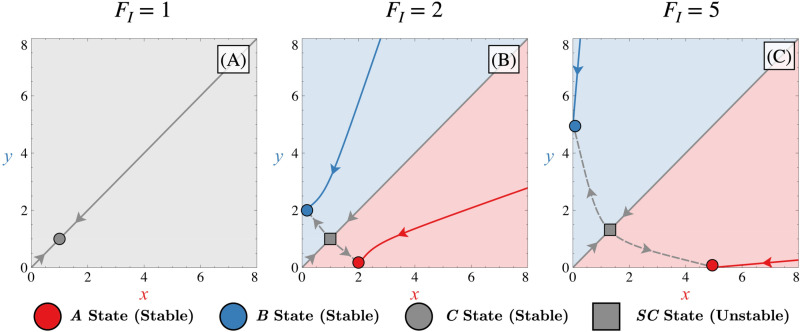
Bistable behaviour. Phase portrait depicting the dynamical system for different values of $F_{I}$ ($F_{I}=1$ (A), $F_{I}=2$ (B), $F_{I}=5$ (C)). In each panel, all $\Delta_{i}=0$, $F_{A}=0$ and κ=0. Corresponding manifolds for states ***A***, ***B*** and ***C*** (in red, blue or grey, respectively) are also represented by solid lines (stable manifolds) or dashed lines (unstable manifolds). These manifolds are computed with the XPP-AUTO software. Different coloured zones in phase space illustrate basins of attraction, leading to trajectories ending in state ***A*** (red), state ***B*** (blue), or remaining non-differentiated in state ***C*** (grey).

The bistable regime can appear in two distinct cases: when the system does not present autoactivations ($F_{A}=0$) and when it does ($F_{A}>0$). In the latter case, the dissociation constant ratio *κ* generally needs to be greater than the autoactivation force $F_{A}$, making the Hill function linked to activation $H_{A}\left(x\right)=F_{A}\frac{x^{n}}{\kappa^{n}+x^{n}}$ much smaller than the Hill function linked to inhibition $H_{I}\left(x\right)=F_{I}\frac{1}{1+x^{n}}$. In both cases, the inhibition force $F_{I}$ (S1 Fig) is the most important parameter affecting the system’s behaviour, as it encodes the interaction between the two transcription factors. Depending on its value, the system can either be monostable (Fig 3A) or bistable (Fig 3B and 3C).

In the bistable regime, trajectories can evolve to one of the two different fates (or states) ***A*** (x≫y) or ***B*** (x≪y), as shown in Fig 3B and 3C. Because we assume a low and symmetric expression of the transcription factors for the initial conditions ($x_{0}=0$, $y_{0}=0$), the system initially evolves along the stable manifold of the non-differentiated state (x≈y), which, in the bistable case, corresponds to a saddle point (referred to as ***SC***). This saddle point plays a pivotal role, as its stable manifold acts as a separatrix in phase space, delineating the boundaries between the basins of attraction for the differentiated states ***A*** and ***B***. Because, in the bistable case, the non-differentiated state is locally unstable, a slight asymmetry in the initial condition or the presence of noise will make trajectories in phase space deviate towards one of the two basins of attraction associated with the differentiated states ***A*** and ***B***. Both stable stationary states are created symmetrically around the separatrix of the phase space when the system becomes bistable and the distance between them increases as $F_{I}$ increases as shown in Fig 3.

The different manifolds observed in Fig 3, which will be discussed throughout this work, were computed using the XPP software [27]. Manifolds represent geometric structures in phase space that guide the trajectories of the system near a stationary state. Intuitively, stable manifolds correspond to directions in phase space along which trajectories asymptotically converge towards a stationary state, while unstable manifolds indicate directions along which trajectories diverge away from it. These manifolds are determined by the eigenvalues and eigenvectors of the Jacobian matrix of the linearized system around the stationary state. In a two-dimensional system such as the one considered here, there are always two eigenvalues, and two corresponding eigenvectors associated with each steady state.

For a saddle point, one eigenvalue is negative and corresponds the stable manifold, which determines the primary direction of convergence, while the other is positive and defines the unstable manifold, guiding the primary direction of divergence in the local vicinity of that state. In contrast, for an attractor, both eigenvalues are negative, and their associated eigenvectors give rise to two stable manifolds that together describe the directions in phase space along which trajectories converge towards the attractor.

When both saddles and attractors coexist, the unstable manifold of the saddle may align with one of the stable manifolds of the attractor. In such cases, trajectories leaving the saddle are guided towards the attractor along this shared manifold, forming a heteroclinic trajectory that links the saddle to the attractor through their respective unstable and stable manifolds (Fig 3B and 3C) [28, 29].

### Tristability in the symmetric case

When the non-differentiated state is represented by a stable node (referred to as ***C***), and in the presence of stable differentiated states ***A*** and ***B***, the system becomes tristable. In the context of cell differentiation, this state represents the co-expression of transcription factors, hindering cell differentiation because of its stability. This transition from the bistable to the tristable regime is also accompanied by the creation of additional unstable states which will change the phase portrait.

**Fig 4:**
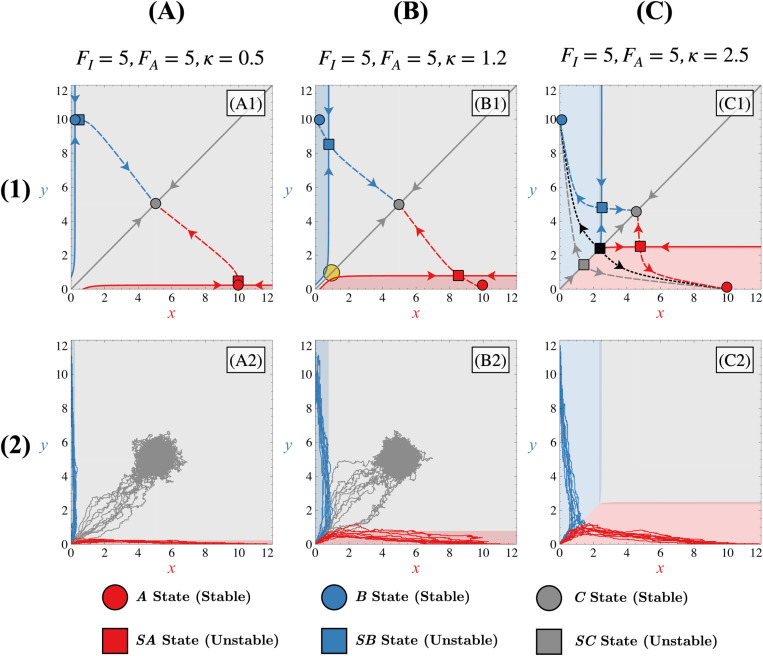
Tristable behaviour. Row (1) panels represent the phase portrait for different tristable regimes (Tristable I regime (column (A)), Tristable II regime (column (C)). The tristable I intermediate case is represented in panels (column (B)). Corresponding manifolds for states ***A***, ***B*** and ***C*** (in red, blue or grey, respectively) are also represented by solid lines (stable manifolds) or dashed lines (unstable manifolds). 10 corresponding trajectories starting from ($x_{0}=0$, $y_{0}=0$) for each case leading to each state are shown in row (2) panels. Different coloured zones in the phase space illustrate basins of attraction, leading to trajectories ending in state ***A*** (red), state ***B*** (blue), or remaining non-differentiated in state ***C*** (grey). In each panel, all $\Delta_{i}=0$, $F_{I}=5$ and $F_{A}=5$. For column (A) panels, κ=0.5; for column (B) panels, κ=1.2; and for column (C) panels, κ=2.5.

As shown in Fig 4, two distinct types of tristable regimes exist and differ in their number of unstable steady states. In a first approximation, we observe that parameter $F_{A}$ has little effect on the system other than allowing tristability. In contrast, for appropriate values of $F_{A}$, the parameter *κ* will determine if the system is in the « Tristable I » case, the « Tristable II » case or even in the bistable case for larger values of *κ*.

For low values of *κ* and for appropriate values of $F_{A}$ (Figs 4A1 and 4A2), the system has three stable nodes (***A***, ***B*** and ***C***) and two saddle points which are located near the differentiated states (***SA*** and ***SB***). We refer to this situation as the « Tristable I » regime. The stable manifolds associated with these new saddle points ***SA*** and ***SB*** represent, for the tristable case, new separatrices of the phase space. Phase space is therefore separated into three different basins of attractions (one for each stable state ***A***, ***B*** or ***C***) and the stable manifold of the non-differentiated state does not act like a separatrix as in the bistable case. Trajectories from low initial conditions can reach the non-differentiated state as shown in Fig 4A2 by following the stable manifold of the non-differentiated stable node ***C***. If there is enough noise in the system, fluctuations will drive trajectories into one of the differentiated states by following one of the stable manifolds associated with the corresponding saddle point ***SA*** or ***SB***. These stable manifolds (corresponding to the ***C***, ***SA*** and ***SB*** states) thus determine the dynamic. The way that trajectories can be distributed into the differentiated states depend on the level of noise in the system and also on the influence of state ***C*** itself. This influence refers to the extent to which the non-differentiated state governs the trajectories originating from low and symmetric initial conditions through its stable manifold. A strong influence means that trajectories tend to move toward state ***C*** rather than populating the differentiated states. Conversely, a weak influence implies that noise in the system allows trajectories to escape the domain of attraction of state ***C***, moving away from its stable manifold. This concept can be interpreted as its ability to influence the velocity field in phase space. In what follows, we refer to this ability of state ***C*** to attract trajectories as the attractivity of this state. Thus, depending on the attractivity of state ***C***, the percentage of trajectories moving toward it will vary. For a highly attractive state ***C***, this percentage can even reach 100%, effectively preventing the cellular differentiation process.

As *κ* increases (Figs 4B1 and 4B2), although the system is still in the tristable I case, the gap between the stable manifolds of the two saddle points ***SA*** and ***SB*** tends to decrease until these manifolds themselves nearly merge. Depending on the noise level or the asymmetry initially present in the initial conditions, this convergence facilitates trajectories to more easily cross one of the two separatrices from low initial conditions, thereby enhancing the efficiency of the differentiation process. When both stable manifolds of these two saddle points are relatively close to each other, we can observe the presence of a saddle-node remnant or « ghost » [29] (Fig 4B1, yellow circle) which has the particularity of influencing the direction of the trajectories of the phase space by attracting them towards it and slowing down their dynamics at this location. This local decrease in the velocity field allows asymmetries/fluctuations to move the trajectory away from the stable manifold of state ***C,*** and thus further populate the differentiated states rather than the non-differentiated one. We can therefore say that the attractivity of the stable non-differentiated state decreases as *κ* increases. Staying within or near this parametric range prevents the non-differentiated state from absorbing all the trajectories starting from low initial conditions.

For higher values of *κ* (Figs 4C1 and 4C2), when the two manifolds are merged, two unstable steady states are created (a saddle point ***SC*** and an unstable node) through a saddle-node bifurcation. In this scenario, trajectories originating from low initial conditions are no longer able to reach the non-differentiated state, as illustrated in Fig 4C2. Indeed, the basins of attraction of ***A*** and ***B*** are now contiguous. Trajectories follow the stable manifold of the saddle point ***SC*** and, once arriving at this point, are pushed back by its unstable manifold of the latter until reaching one of the differentiated states ***A*** or ***B***. Under these conditions, it is therefore the stable manifold of the newly created saddle point ***SC*** which now acts as a separatrix. It will entirely determine the dynamics of the system for low initial conditions, forcing trajectories to differentiate and thus making this new configuration similar to the bistable one. We label this new configuration as the « Tristable II » regime. If *κ* increases further, the stable state ***C*** disappears through a pitchfork bifurcation that merges the ***SA***, ***SB*** and ***C*** states, creating a saddle state. This saddle state is immediately annihilated by a saddle-node bifurcation involving this saddle and the unstable node (S2 Fig). At this stage, only the differentiated states ***A***, ***B*** and the unstable non-differentiated state ***SC*** remain, resulting in a bistable system (if the $F_{I}$ value is sufficiently large) as the original Toggle Switch, despite the presence of autoactivations within the system.

The fact that *κ* plays a key role in the stability regime of the system and the reachability of the non-differentiated state can be explained as follows. When *κ* is low, the autoactivation Hill function $H_{A}\left(x\right)=F_{A}\frac{x^{n}}{x^{n}+\kappa^{n}}$ tends to $F_{A}$. In this case, the autoactivation term can be, for a sufficiently large value of $F_{A}$, larger than the inhibition Hill function $H_{I}\left(x\right)=F_{I}\frac{1}{1+x^{n}}$ and the evolution equations (9)-(10) take the approximate forms $\frac{dx}{dt}\approx F_{A}-x$ and $\frac{dy}{dt}\approx F_{A}-y$. This system of equations admits only one stable steady state and represents the extreme case where the non-differentiated state is totally attractive. The inhibition term has then less impact on the dynamics and cannot favour any specific state, even if one of the differentiated states is favoured by the GRN. This is why, for very low values of *κ*, all trajectories converge to the non-differentiated state. We observe that, despite differences in the construction of model equations—specifically, the additive implementation of autoactivations in our approach versus the multiplicative implementation in reference [2]—our findings align with those of Jia et al. [2]. Both studies demonstrate that strong autoactivations enhance the stability of the non-differentiated state ***C*** at the expense of the differentiated states (***A*** and ***B***). This consistency across different approaches suggests that this effect could be an intrinsic property of this type of regulation between transcription factors. As *κ* increases, the function $H_{A}\left(x\right)$ no longer tends to $F_{A}$ but tends to 0 if *κ* is quite large. This translates into the fact that the non-differentiated stable state ***C*** becomes progressively less accessible from low initial conditions (Figs 4A1, 4A2, 4B1 and 4B2) until the system becomes tristable.

### Effect of asymmetries on multistability

#### Bistable case.

While the previous section focused on the behaviour of the GRN in a symmetric case, in this section we consider the different cases where asymmetries are present and focus on the effect of these asymmetries on the phase portrait.

In the bistable regime, the parameters contributing to asymmetry are exclusively $\Delta_{FI},\Delta_{KI}$ and $\Delta_{D}$. This is because, in this regime, large values of *κ* render the autoactivation terms $H_{A}$ (Eqs (9) and (10))—and consequently, their associated asymmetry parameters—negligible compared to those linked to inhibition, as explained in the previous section.

**Fig 5:**
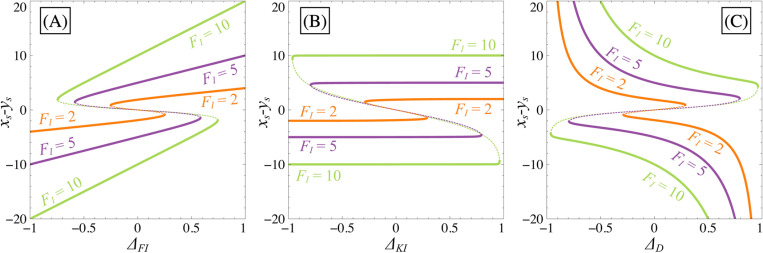
Effect of asymmetries on bistability. Bifurcation diagrams illustrating the influence of asymmetry parameters on the system (9)-(10). Panels (A), (B) and (C) represents variations in $\Delta_{FI}$, $\Delta_{KI}$, and $\Delta_{D}$, respectively, while $F_{I}$ is fixed at different values (2 (orange), 5 (purple) and 10 (green)). In each panel, all $\Delta_{i}=0$ except for the one that changes, $F_{A}=0$ and κ=0. Stable branches are depicted by continuous lines, while unstable branches are represented by dotted lines.

**Fig 6:**
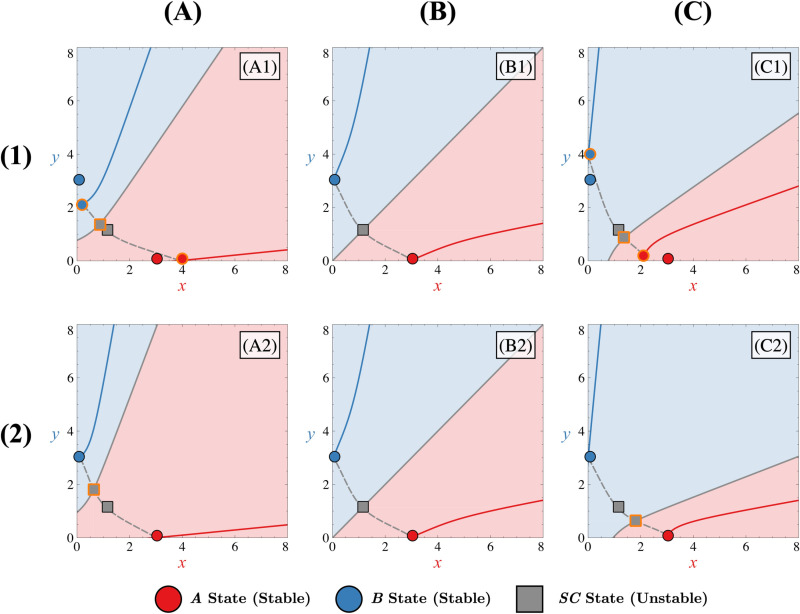
Effect of asymmetries on the bistable phase portrait. Shift of steady states when asymmetry favours state ***A*** (column (A), Δ=0.1) or state ***B*** (column (C), Δ=−0.1) compared to the symmetric case (column (B), Δ=0). Asymmetry applied through $\Delta_{FI}$, $\Delta_{KI}$ corresponds to rows (1) and (2) respectively. In each panel, $F_{I}=3$, $F_{A}=0$, κ=0 and all $\Delta_{i}=0$ except for the one that change. Corresponding manifolds for states ***A***, ***B*** and ***C*** (in red, blue or grey, respectively) are also represented by solid lines (stable manifolds) or dashed lines (unstable manifolds). Different coloured zones in the phase space illustrate basins of attraction, leading to trajectories ending in state ***A*** (red), state ***B*** (blue), or remaining non-differentiated in state ***C*** (grey). In panels corresponding to an asymmetric case (A1, A2, C1, C2), both sets of steady states in the symmetric and asymmetric case are represented with a black or orange contour, respectively.

Bifurcation diagrams in Fig 5 show that the bistability can be lost depending on the level of asymmetries through a saddle-node bifurcation, regardless of the nature of asymmetry. The ability of the system to maintain bistability depends on the strength of the inhibition $F_{I}$. For larger values of $F_{I}$, the distance between the two limit points of the saddle-node tends to be larger making the bistable domain and the distance between the two differentiated states larger as well. These diagrams also show that the shift of the steady states depends on the nature of the asymmetry itself. Indeed, we can see that introducing an asymmetry at the dissociation constant level (by changing $\Delta_{KI}$) will not impact the position of the differentiated states in phase space (Fig 5B) which is not the case if asymmetry is at the transcription speed or degradation level (Figs 5A, 5C and 6). Nevertheless, regardless of the asymmetry type, the position of the non-differentiated ***SC*** will always change.

Fig 6 illustrates the displacement of states in phase space and highlights that the separatrix position can change independently of the position of the differentiated states. In other words, the separatrix is only determined by the position of the non-differentiated state, influencing the evolution of trajectories towards the differentiated states. There exists then a direct correlation between the saddle point and the separatrix positions. If the separatrix shifts significantly due to asymmetry, trajectories from low initial conditions will predominantly reach the favoured state. Because the steady state position of the unstable non-differentiated state changes regardless of the nature of the applied asymmetry, the bistable case is highly sensitive to asymmetries in the GRN.

Approximate expressions for the steady-state values of $x_{s}$ and $y_{s}$ for each state are provided in S2 Table.

#### Tristable case.

Given the number of possible asymmetries ($\Delta_{FI},\Delta_{FA},\Delta_{KI},\Delta_{KA}$ or $\Delta_{D}$) and possible parametric configurations (given by $F_{I},F_{A}$ and *κ*) for the tristable regimes, we restricted our analysis to the two main regimes discussed in the previous section (tristable I and tristable II) and looked at how asymmetries impact the phase portrait in a general way. The different bifurcation diagrams of the system depending on each source of asymmetry and the approximative expression for $x_{s}$ and $y_{s}$ for each state are provided in the supplementary materials (S3 Fig and S2 Table respectively). In both tristable scenarios, given that the phase space is now delimited by the stable manifolds associated with the saddle points ***SA*** and ***SB***, it is no longer relevant to only know the position of the non-differentiated state ***C*** to predict how separatrices will change and, therefore, which differentiated state will be favoured by an asymmetry in the GRN.

In the tristable I scenario, our observations indicate that the change in the location of the separatrices, when asymmetries are introduced, is connected to the attractivity of the non-differentiated state. Depending on the attractivity of the non-differentiated state, the differentiated states and their associated saddle points are more or less close in the phase space, as indicated in Figs 4A1 and 4B1. This proximity between these states in phase space has an impact on how these states depend on asymmetries and, consequently, on how the separatrices depend on asymmetries.

**Fig 7:**
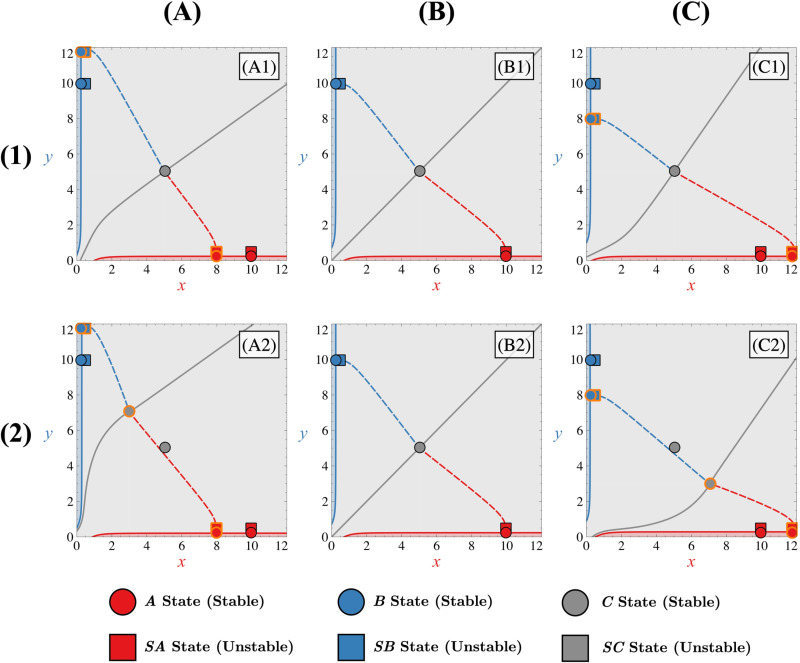
Effect of asymmetries on tristable phase portrait with a highly attractive non-differentiated state. Shift of steady states when asymmetry favours state ***A*** (column (A), Δ=0.4) or state ***B*** (column (C), Δ=−0.4) compared to the symmetric case (column (B), Δ=0). Asymmetry applied through $\Delta_{FI}$, $\Delta_{FA}$ corresponds to rows (1) and (2) respectively. In each panel, $F_{I}=5$, $F_{A}=5$, κ=0.5 and all $\Delta_{i}=0$ except for one that change. Corresponding manifolds for states ***A***, ***B*** and ***C*** (in red, blue or grey, respectively) are represented by solid lines (stable manifolds) or dashed lines (unstable manifolds). Different coloured zones in the phase space illustrate basins of attraction, leading to trajectories ending in state ***A*** (red), state ***B*** (blue), or remaining non-differentiated in state ***C*** (grey). In panels corresponding to an asymmetric case (A1, C1, A2, C2), both sets of steady states in the symmetric and asymmetric case are represented with a black or orange contour, respectively.

When the non-differentiated state is highly attractive (Fig 4A1), the differentiated states are close to their associated saddle points (***A*** with point ***SA*** and ***B*** with point ***SB***). This proximity in phase space leads to similar mathematical expressions for their steady-state values and, consequently, their dependence on asymmetries (S3 Fig). Hence, these states tend to move similarly in the presence of asymmetries, regardless of their nature. This results in a locally less altered velocity field, rendering the separatrices less sensitive to the imposed asymmetries, as illustrated in Fig 7.

Regarding the stable non-differentiated state ***C***, while its displacement caused by asymmetries may affect its stable manifold (Figs 7A2 and 7C2), its impact on the separatrices can be considered to be negligible compared to the case where its position remains unchanged (Figs 7A1 and 7C1). Moreover, if the non-differentiated state is highly attractive, the separatrices remain relatively distant from the point (0,0) in phase space. This implies that significant asymmetry is needed before trajectories from low initial conditions favour one of the differentiated states. In such conditions, the stable manifold of the non-differentiated state guides phase space trajectories emanating from weak initial conditions, which do not directly fall into a basin of attraction of differentiated states.

Another important consequence of this proximity between the differentiated states and their respective saddle points is that they cannot annihilate each other via a saddle-node bifurcation (S3 Fig). This is, again, because they consistently displace in the same direction without meeting, regardless of the type of asymmetry present in the system. Therefore, the only way for the system to annihilate tristability, in cases where the non-differentiated state is highly attractive, is for this state itself to be moved by certain asymmetries towards one of the saddle points. However, as indicated in the S2 Table and S3 Fig, the only asymmetries capable of inducing movement of the ***C*** state and thereby altering the system’s stability are those linked to the autoactivations and degradation ($\Delta_{FA}$, $\Delta_{KA}$ and $\Delta_{D}$). This conclusion is further supported by an analysis of the derivatives of the steady-state expressions for state ***C*** (S2 Table) with respect to the asymmetry parameters, which show non-zero values only for $\Delta_{FA}$, $\Delta_{KA}$ and $\Delta_{D}$. This suggests that asymmetries at the level of inhibitory interactions cannot alter the system’s regime. Consequently, it can be inferred that external signalling that only affects the inhibitory interactions between genes would not impact the system’s multistability. This highlights the distinct roles that autoactivation and degradation asymmetries play in driving regime changes, in contrast to inhibitory asymmetries which appear limited in their capacity to influence stability.

**Fig 8:**
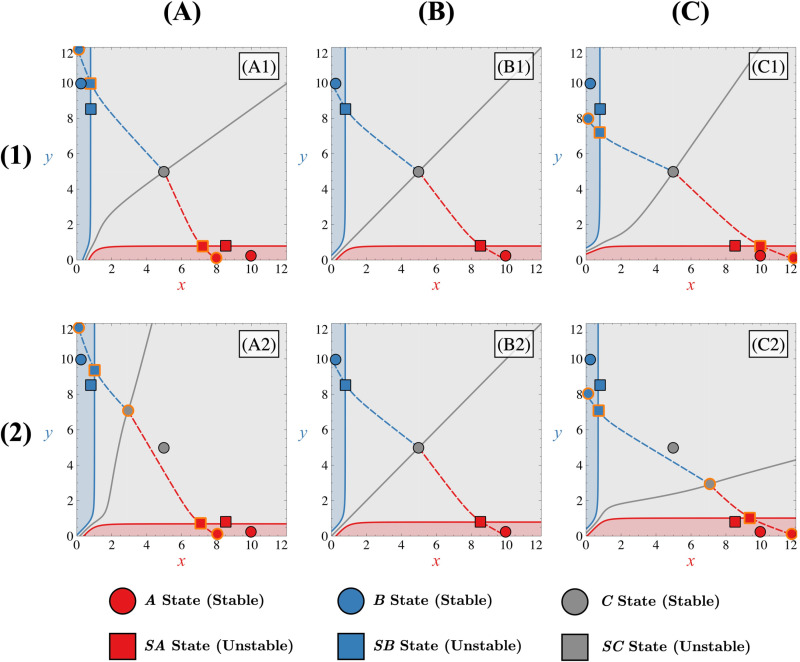
Effect of asymmetries on tristable phase portrait with a poorly attractive non-differentiated state. Shift of steady states when asymmetry favours the ***A*** state (column (A), Δ=0.4) or the ***B*** state (column (C), Δ=−0.4) compared to the symmetric case (column (B), Δ=0). Asymmetry applied through $\Delta_{FI}$, $\Delta_{FA}$ corresponds to rows (1) and (2) respectively. In each panel, $F_{I}=5$, $F_{A}=5$, κ=1.2 and all $\Delta_{i}=0$ except for one that change. Corresponding manifolds for states ***A***, ***B*** and ***C*** (in red, blue or grey, respectively) are represented by solid lines (stable manifolds) or dashed lines (unstable manifolds). Different coloured zones in the phase space illustrate basins of attraction, leading to trajectories ending in state ***A*** (red), state ***B*** (blue), or remaining non-differentiated in state ***C*** (grey). In panels corresponding to an asymmetric case (A1,C1,A2,C2), both sets of steady states in the symmetric and asymmetric case are represented with a black or orange contour, respectively).

When the attractivity of the non-differentiated state decreases (Fig 4B1), the differentiated states and their saddle points tend to move away from each other. This causes the differentiated states and their respective saddle points to exhibit different dependencies on asymmetries (S3 Fig). Consequently, the distances between these points can change when asymmetries are applied to the system, which contrasts with the behaviour observed in the highly attractive case. This separation leads to a local imbalance in the velocity field and, consequently, influences the separatrices, as depicted in Fig 8. For instance, as depicted in Fig 8A1, compared to the symmetric scenario (Figs 8B1 or 8B2), the distance between differentiated state ***A*** and its saddle point ***SA*** decreases, while it increases between differentiated state ***B*** and its saddle point ***SB*** (due to anti-asymmetry on both variables *x* and *y*). Consequently, the separatrix associated with state ***B*** shifts towards the origin (0,0), while the separatrix associated with state ***A*** moves away from it. In such a scenario, the differentiated state ***B*** is favoured, as trajectories originating from low initial conditions tend to converge towards this state. In contrast to the highly attractive case, the movement of state ***C*** significantly influences the velocity field. Specifically, depending on the direction in which asymmetries displace state ***C*** in phase space, this state can either reinforce or weaken the imbalance in the velocity field thus making separatrices more or less sensitive to asymmetries. For instance, in the scenario where asymmetries favour state ***A*** (Figs 8C1 and 8C2), we observe that the impact of the different displacement of differentiated states and their saddle points on the velocity field is offset by the movement of state ***C*** toward the favoured state ***A*** (Fig 8C2). As a result, the displacement of the separatrix associated with the saddle point ***SA*** towards the origin is reduced. The sensitivity of this separatrix to asymmetries is therefore reduced, allowing trajectories originating from low initial conditions to remain non-differentiated at the beginning of the evolution of the system. Otherwise, in cases where state ***C*** is not displaced by asymmetries (Fig 8C1), trajectories for low initial conditions are predetermined to evolve towards state ***A***.

Moreover, when the differentiated states and their saddle points are far apart, the separatrices tend to be close to the point (0,0) for a symmetric system (Figs 8B1 or 8B2). In this case, applying asymmetries to the system (Figs 8A1, A2, C1 or C2) may cause the separatrices to exceed this point, and trajectories from weak initial conditions may entirely start within one of the two attraction basins associated with the differentiated states, leading them to converge into one of the two differentiated states. The stable manifold of the non-differentiated state no longer entirely dominates the dynamics of trajectories emanating from weak initial conditions.

Regarding tristability, our observations reveal that it still cannot be modified by bifurcations between the differentiated states and their associated saddle points (S3 Fig). However, since these same saddle points are closer to the non-differentiated state in phase space, a lower level of asymmetry is needed for the saddle points to collapse with the non-differentiated state compared to when this state is highly attractive. Once again, this reveals that the attractivity of the non-differentiated state plays a predominant role not only in the sensitivity of the separatrices but also in the robustness of the tristable regime.

For the tristable II case, the appearance of a new saddle point ***SC*** along the bisector of the phase space near low values of *x* and *y* will make the behaviour of the system in the presence of asymmetries similar to that of the bistable case (Fig 4C). Indeed, it is now the position of this saddle point ***SC*** (grey square in Fig 4C1) and then the position of its corresponding stable manifold (grey solid line attached to the grey square in Fig 4C1) that will control the fact that a trajectory can reach or not one of two differentiated states. This stable manifold is then the separatrix of the phase space for low initial conditions. Therefore, this indicates that any asymmetry that changes the position of this saddle point will also change the position of the separatrix for low initial conditions and thus how the trajectories will go or not towards one of the two differentiated states.

Finally, in the same way as in the bistable case, the sensitivity of the separatrices is influenced by the values of $F_{I}$ or $F_{A}$, irrespective of whether the system is in the tristable I or tristable II regime. A higher $F_{I}$ or $F_{A}$ value results in increased separation between steady states, leading to a greater separation between the differentiated states and their corresponding saddle points from the (0,0) point. Consequently, the separatrices require more asymmetries in the system to reach the (0,0) point or, in other words, to preferentially favour a differentiated state.

### Robustness of the differentiation process

As mentioned in the introduction, an important feature of the present model is its ability to replicate the observed robustness in key properties of the differentiation process [30–34]. This section delves into the analysis of two such properties. The first concerns the probability of selecting a specific differentiated state, which is examined by assessing the proportions of stochastic trajectories from low initial conditions leading to states ***A***, ***B*** and ***C***. The second one involves the capability of the trajectory (or the cell) to maintain this choice. For the latter, we will look at the ability of a stochastic trajectory to transition between states due to inherent noise in the system after cells become differentiated.

**Fig 9:**
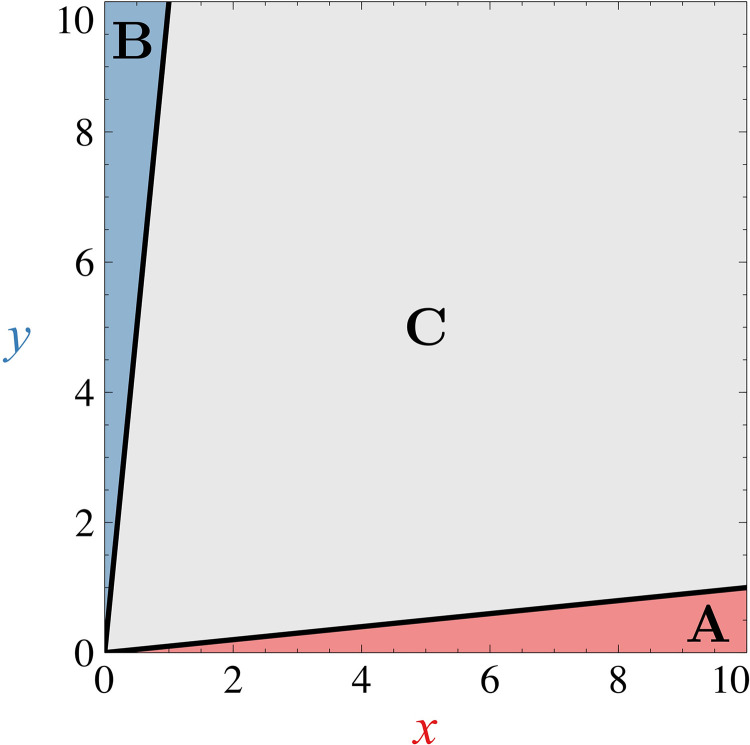
Spatial coarse graining of phase space. Black lines represent the different boundaries between each coarse-grained state and are given by Eqs (11) and (12). In this case, $F_{I}=0$. Trajectories that are in the red, blue or grey domains corresponds to ***A***, ***B*** or ***C*** cells respectively.


To analyse various properties concerning the state of a cell, it is essential to discretize the phase space into three coarse-grained domains, corresponding to the basins of attraction of the stable ***A***, ***B*** or ***C*** states. Given the difficulty of computing the different separatrices of the phase space, we decided to simplify the computational task of discretizing the phase space by using the following conditions which are represented in Fig 9:

$$\text{If}x>10y+\frac{F_{I}\left(1+\Delta_{FI}\right)}{2\left(1+\Delta_{D}\right)}\text{then the system is in the }A\;\text{state,}$$ (11)

$$\text{If}y>10x+\frac{F_{I}\left(1-\Delta_{FI}\right)}{2\left(1-\Delta_{D}\right)}\;\text{then the system is in the }B\;\text{state,}$$ (12)

and if none of these conditions are met, then the system is in the ***C*** state.

This choice is motivated by the location of the different steady states (S2 Table) and an examination of stationary probability distributions for various parametric conditions. Furthermore, this coarse graining is intended to be applicable regardless of the system’s regime. In the tristable case, each of the three domains corresponds to a true basin of attraction, as all three states (***A***, ***B***, and ***C***) are stable. However, in the bistable case, the non-differentiated state ***SC*** is unstable and therefore does not possess a basin of attraction. Despite this, we maintain the ***C*** domain in the coarse-grained discretization to provide a consistent framework that allows us to observe the dynamics of trajectories passing through this region. For bistable configurations, any trajectory entering the ***C*** domain will eventually return to one of the stable states, ***A*** or ***B***, as it does not encounter a true stable state within the ***C*** domain. This consistent three-domain discretization allows us to compare different parametric configurations regardless of the presence or the absence of autoactivations (which determine if the system is bistable or tristable). Therefore, the discretization depends solely on parameters influencing the position of stationary states in the case without autoactivations (specifically parameters $F_{I}$, $\Delta_{FI}$ and $\Delta_{D}$, excluding $F_{A}$, *κ*, $\Delta_{FA}$ and $\Delta_{KA}$).

For each property, we assess the influence of parameter values to establish a correlation between the structure of the GRN and these properties. Subsequently, we explore the impact of the non-differentiated state on these properties by comparing the various regimes defined in the preceding section, namely the bistable, tristable I and tristable II cases. Furthermore, we investigate two different values of the extensivity parameter Ω (10 and 100) to understand the implications of system size and fluctuations. Finally, given the different possible sources contributing to asymmetry in the system, we conduct individual analyses for each source to elucidate their specific impact on these properties.

#### Cell fate decision.

The choice of a cell to go in one of the differentiated states is a highly reproducible mechanism within the biological system experiencing differentiation [31–34]. This phenomenon takes place despite stochastic evolution in the concentrations of transcription factors, due to intrinsic noise or despite asymmetries that are inherently present in the GRN. In this section, our goal is to identify parametric configurations (determined by $F_{I},F_{A}$ and *κ*) that result in the system maintaining an equilibrium in the fraction of trajectories reaching differentiated states ***A*** and ***B*** when asymmetries are introduced. Essentially, we aim to understand the extent of parametric asymmetries that can be introduced into the initially symmetric network before one differentiated state becomes significantly favoured over the other. A parametric configuration demonstrating constancy in the differentiated state’s ratio under the influence of asymmetries will be referred to as « robust ».

**Fig 10:**
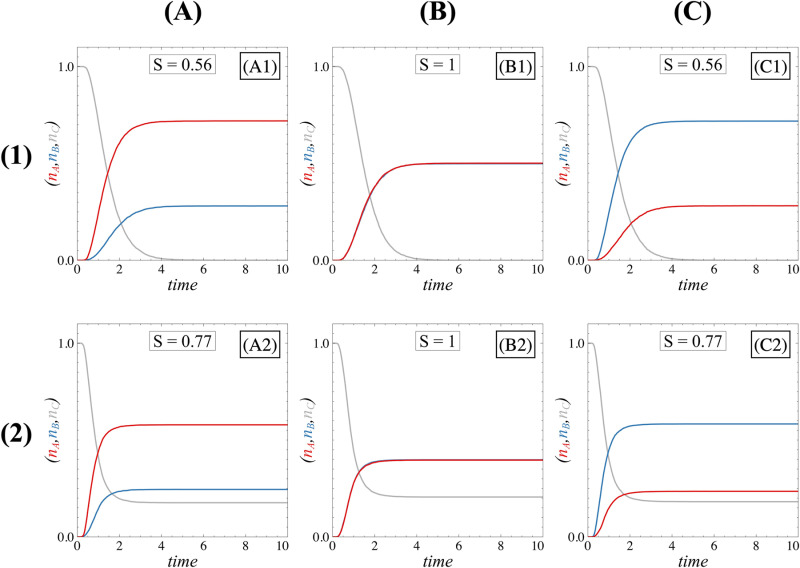
Time evolutions of proportions. Time evolutions of the proportions of trajectories occupying states ***A*** ($n_{A}$, red curve), ***B*** ($n_{B}$, blue curve), or ***C*** ($n_{C}$, grey curve). Column (A) panels and column (C) panels depict the time evolutions for an asymmetric GRN (Δ=0.1, Δ=−0.1, respectively) while column (B) panels stand for a symmetric, in both bistable (or tristable II) (row (1)) and tristable I (row (2)) scenarios. In all panels, $F_{I}=5$. For row (1) panels, $F_{A}=0$ and κ=0; for row (2) panels, $F_{A}=5$ and κ=1.5. For column (A) and (C) panels, the asymmetry is applied through $\Delta F_{I}$ which is equal to 0.1 and −0.1, respectively. The system size Ω is equal to 10. Proportions were calculated as fractions of trajectories ending ***A***, ***B*** or ***C*** domain defined by our coarse graining for 10.000 Gillespie simulations.

To this end, for each parametric condition, we computed the fraction of stochastic trajectories leading to the coarse-grained region associated with the ***A*** state (denoted as $n_{A}$), the ***B*** state ($n_{B}$) and the fraction of stochastic trajectories that remain within the non-differentiated region ($n_{C}$). All these quantities evolve over time and converge at the steady state to a given value as shown in Fig 10 for the cases where the system is bistable or tristable. In the symmetric case and for a symmetric low initial condition ($x_{0}=0$, $y_{0}=0$), the proportions of differentiated cells are obviously equal. If the system is bistable, there is a 50% chance to go in each differentiated state (Fig 10B1) and $n_{A}=n_{B}=0.5$ while $n_{C}=0$. For the tristable case (Fig 10B2), the equality $n_{A}=n_{B}$ remains true but the proportions $n_{A}$ and $n_{B}$ are not equal to 0.5. This is because, in this case, some cells can remain non-differentiated and $n_{C}>0$. If we now add asymmetry to the system, this equality in the proportions of the differentiated states is lost (Figs 10A1, 10A2, 10C1 and 10C2).

For a given level of asymmetry and a given parametric condition, the amplitude of the deviation from equal proportions of ***A*** and ***B*** states can be estimated by taking the absolute difference between $n_{A}$ and $n_{B}$ at the steady state. Subsequently, we introduce the « symmetry measure » $S=1-\left|n_{A}-n_{B}\right|$, which is equal to 1 for equal proportions of cell types (symmetric GRN), and smaller than 1 otherwise (asymmetric GRN). For a given parametric configuration ($F_{I},F_{A},\kappa$), by computing the mean $\Sigma_{S}=\frac{1}{N}\overset{\Delta =1}{\sum_{\Delta =-1}}S\left(\Delta \right)$ where *N* is the number of tested values of Δ, we gain insights into the sensitivity of differentiated cell proportions relative to the symmetric case. $\Sigma_{S}=1$ indicates that proportions of differentiated cells consistently maintain the same value and the proportions are fully robust against asymmetries. Conversely, $\Sigma_{S}\to 0$ means that proportions strongly depend on asymmetries.

**Fig 11:**
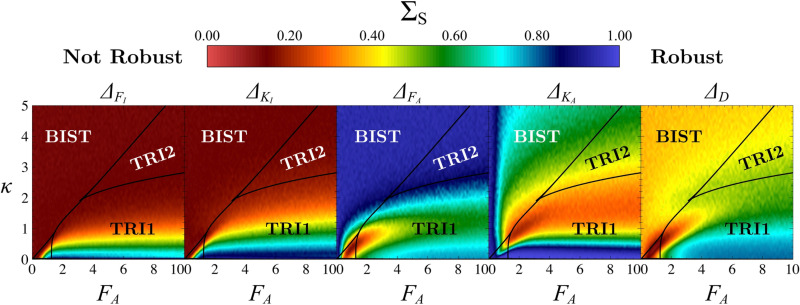
Robustness diagrams in parametric space. Parametric space spanned by $F_{A}$ and *κ* for different types of asymmetries taken one by one. $F_{I}$ is fixed at 5, and the system size is Ω=10 (other values of $F_{I}$ and Ω are provided in S3 Fig). The colour scale represents the $\Sigma_{S}$ value at the steady state which is the mean of S over the entire domain of possible values for only one given asymmetry $(\Delta_{i}=-1$ to $\Delta_{i}=1$). Red zones ($\Sigma_{S}\approx 0$) indicate less robust cases, while blue zones ($\Sigma_{S}=1$) denote the most robust parametric regions with respect to the asymmetry which is considered. For each ($F_{A},\kappa$), $\Sigma_{S}$ is computed at the steady state from 200 Gillespie simulations. The system is considered stationary after 20-time units. Stability domains are the same as in Fig 2. Simulations were performed in the parametric space with a discretization step of 0.1 for *κ* and $F_{A}$.

In Fig 11, we present the values of $\Sigma_{S}$ at the steady state within the parametric space spanned by $F_{A}$ and *κ*, for a fixed value of $F_{I}=5$ and Ω=10. These graphs, referred to as robustness diagrams, offer insight into various general trends, which we will elaborate on in the following. Additional robustness diagrams for different $F_{I}$ and Ω are provided in S4 Fig as well as the graphs for the proportions of ***A***, ***B*** and ***C*** in the symmetric case in S5 Fig and S6 Fig.

**Fig 12:**
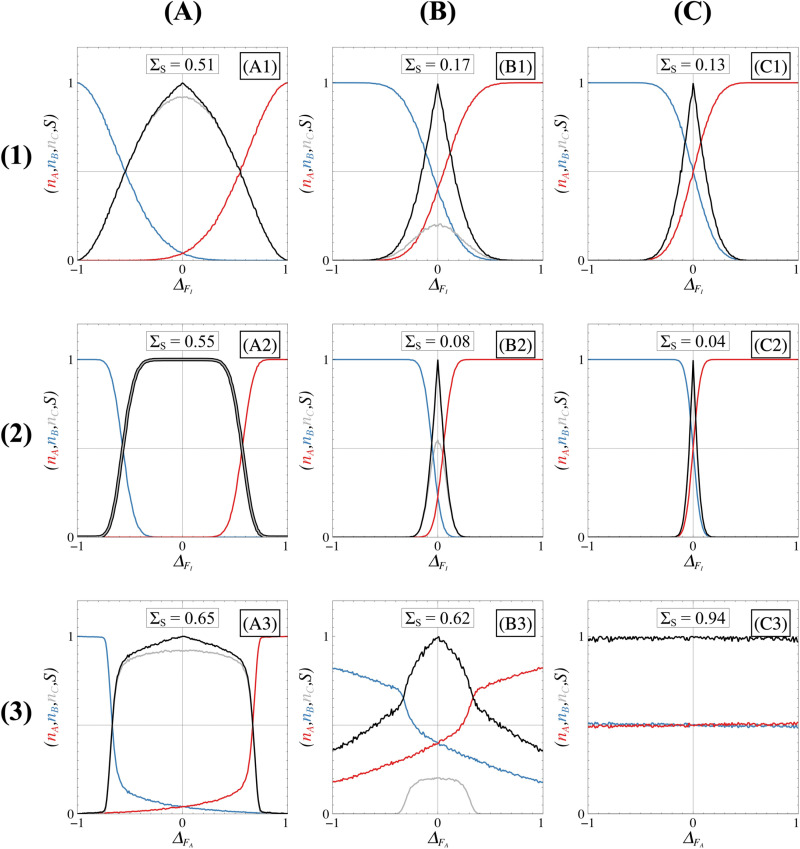
Impact of regime on asymmetry-dependent steady state proportions. Proportions of ***A*** cells (red), ***B*** cells (blue), and non-differentiated ***C*** cells (grey) as a function of the asymmetry level $\Delta_{FI}$ (rows (1)-(2)) and $\Delta_{FA}$ (row (3)). Three different regimes are shown: the tristable I where ***C*** is highly attractive (column (A)), tristable I where ***C*** is poorly attractive (column (B)), and bistable or tristable II (column (C)). Black curves represent the symmetry measure, $S=1-\left|n_{A}-n_{B}\right|$, with spreads reflecting the $\Sigma_{S}$ value (noted in each panel). In all panels, $F_{I}=5$ and $F_{A}=5$ but the value of *κ* changes: column (A) panels use κ=0.5, column (B) panels use κ=1.5, and column (C) panels use κ=5.0. Rows (1)-(3) and row (2) correspond to system sizes Ω=10 and Ω=100, respectively. Proportions were calculated as fractions of trajectories ending in ***A***, ***B*** or ***C*** domains defined by our coarse graining for 10.000 Gillespie simulations. The system is considered stationary after 20-time units.

In Fig 12A1, for a highly attractive non-differentiated state and given our definition of robustness, *S* slowly decreases when asymmetries are applied to the system, establishing high robustness of the proportions. While this highly attractive case ensures robustness, it does not allow for cell differentiation given that the proportions of ***A*** and ***B*** are extremely low (see S5 Fig and S6 Fig). As the non-differentiated state proportion diminishes, the spread of the *S*-curves diminishes (equivalent to the $\Sigma_{S}$ value), which signifies a decrease in robustness. Comparison between these cases shows that the bistable scenario, regardless of *κ*, $F_{I}$ and $F_{A}$ values, corresponds to the least robust case but ensures a total differentiation of cells.

These observations lead directly to the major conclusion that the presence of the non-differentiated state can reinforce proportions robustness, even though it hinders trajectories from reaching a differentiated stable state when excessively attractive. This phenomenon is explained by the limited movement of the separatrices when the non-differentiated state exhibits high attractivity as explained in the previous section. It is therefore important to carefully choose a parametric configuration when the non-differentiated state is attractive but not too much to avoid that cells remain non-differentiated.

Noise also contributes to the robustness of the proportions against asymmetries. Indeed, by comparing Figs 12A2 and 12B2 or Figs 12A3 and 12B3, we observe that the spread of the *S*-curves decreases as the system size increases, indicating a decrease in the robustness of the proportions. This is because fluctuations can lead trajectories into the basin of attraction associated with the differentiated state disadvantaged by the asymmetries. Additionally, comparing Figs 12A2 and 12B2 reveals that stochastic noise reduces the attractivity of the non-differentiated state. This happens because stochastic noise allows trajectories to leave the stable manifold of the non-differentiated state (when stable) and can guide them toward one of the basins of attraction associated with a differentiated state. Consequently, noise enhances the efficiency of the differentiation process.

By comparing Figs 12A1, 12B1 and 12C1 with Figs 12A3, 12B3 and 12C3, we show that the nature of the asymmetries themselves influences the way the system responds to asymmetries. The behaviours observed are attributed to whether the positions of steady states depend on specific asymmetries, as indicated in the S2 Table. For example, in the bistable case, the location of the saddle point ***SC*** governing the position of the separatrix depends on the $\Delta_{FI}$ asymmetry, but not on $\Delta_{FA}$ (S3 Fig), which explains the independence of proportions in Fig 12C3. A closer examination of Fig 11 reveals that asymmetries linked to inhibition significantly influence proportions for each regime, highlighting the fundamental role of inhibitions. As for asymmetries related to autoactivations, their impact on sensitivity appears less pronounced than the asymmetries linked to inhibition in the tristable I regime until it becomes entirely independent in the bistable or tristable II case.

### Transitions between cell fates

**Fig 13:**
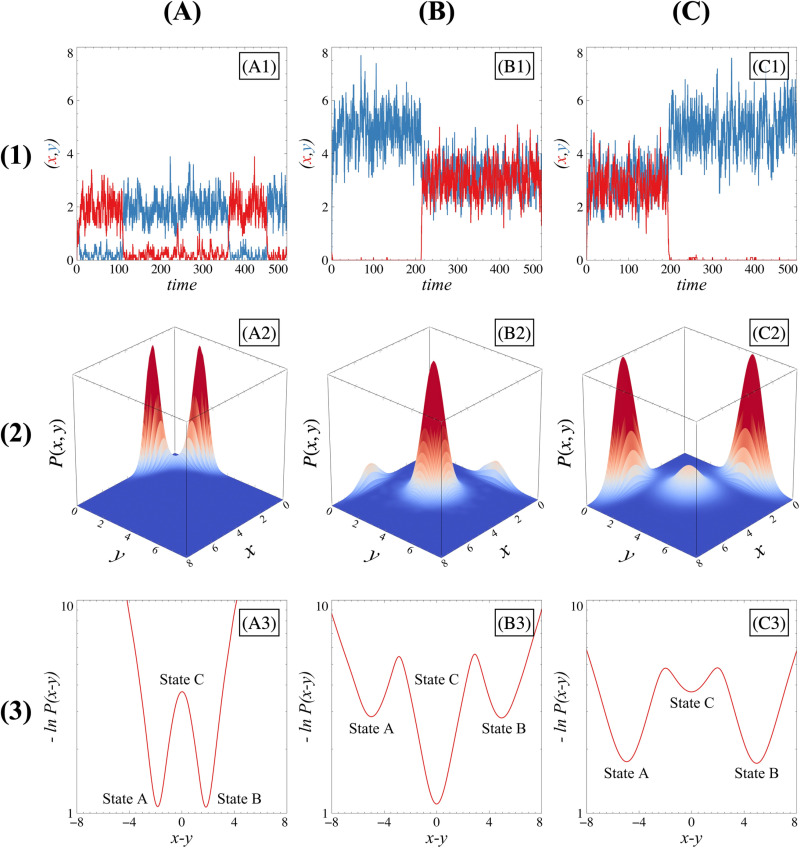
Transitions between states for different scenario. Each column (A), (B) or (C) indicates a different transition scenario: A⇄B (column (A)), *A* or B→C (column (B)), C→A or *B* (column (C)). Row (1) panels represents time evolution trajectories of *x* (red lines) and *y* (blue lines) concentrations. Row (2) panels display the steady state probability distributions as a function of macroscopic variables *x* and *y*. These distributions are computed from 10.000 simulations. Row (3) panels display kinetic potentials at the steady state as a function of the variable x−y. These potentials are calculated based on the corresponding steady-state probability distributions, obtained through the Gillespie algorithm over 100.000 simulations, which are then smoothed into continuous distributions using kernel density estimation. The system is considered stationary after 20-time units. Il all scenarios, $F_{I}=2$. For the first scenario (A⇄B column (A)), $F_{A}=0$ and κ=0; for the second scenario (*A* or B→C, column (B)), $F_{A}=3$ and κ=0.4; and for the third scenario (C→A or *B*, column (C)), $F_{A}=3$, κ=1.2. For each case, the extensivity parameter Ω=10.

Another crucial aspect of the differentiation process is the necessity for cells to persist in their chosen states over a sufficient period of time. Experiments show that transitions between differentiated states (***A*** to ***B*** or ***B*** to ***A***) and transitions from a differentiated state to a non-differentiated state (***C*** to ***A*** or ***B***) do most of the time not occur spontaneously [30,35–38]. Consequently, such transitions should also be precluded in theoretical models for cell differentiation. We start by assessing their occurrence in the case of symmetric GRNs. Because we adopt a stochastic description, fluctuations can induce transitions [39] (Figs 13A1, 13B1 and 13C1) between states when the probability distribution of *x* and *y* is multimodal (Figs 13A2, 13B2 and 13C2) in the system considered here.

#### Symmetric case.

Steady-state kinetic potentials $U\left(x,y\right)=-lnP_{s}\left(x,y\right)$ derived from probability distributions at the stationary state serve as valuable tools to address the challenge of studying transitions [38,40–49]. However, the focus of this work is on transitions between states that differ in their value z=x−y. Therefore, the results presented below all correspond to the reduced kinetic potential $U\left(z\right)=-lnP_{s}\left(z\right)$. It should be noted that a Principal Component Analysis (PCA) performed on the steady-state values of *x* and *y* reveals that *z* is the first principal component of our system, which further confirms the relevance of focusing on this variable.

Figs 13A3, 13B3 and 13C3 show the resulting potentials for different parametric configurations. In the symmetric bistable case (Fig 13A3), the non-differentiated state ***SC*** is unstable, and the system will not remain in this state during transitions. Moreover, a transition starting from one differentiated stable state (say, state ***A***) and crossing the stable manifold of the unstable state ***SC*** will result in a transition to the opposite differentiated state (***B***), since the unstable manifold corresponding to state ***SC*** will force the phase-space trajectories into this direction. Consequently, transition sequences A→C→B or B→C→A effectively result in reversible transitions of type A⇄B.

In the tristable case (Figs 13B3 and 13C3), fully reversible transition sequences of the type A⇄C⇄B can be observed because the non-differentiated state ***C*** is now stable. The stability of ***C*** enables the system to not only reach and remain in this state (A→C or B→C) but also to return to its initial state before the transition, thereby making transitions such as A→C→A or B→C→B possible. Therefore, having a tristable system intuitively decreases the probability of observing trajectories switching from one differentiated state to another.

**Fig 14:**
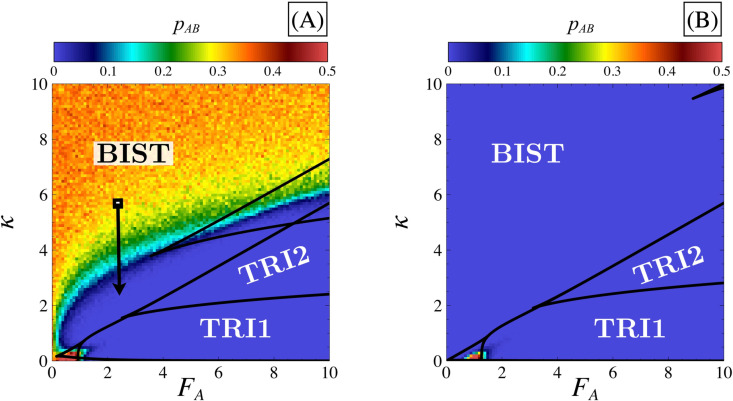
Transition probability between A and B states. Panels (A) and (B) display the transition probability $p_{ab}$ between states ***A*** and ***B*** in the parametric space $F_{A},\kappa$ for different values of $F_{I}=2$ (panel A) and $F_{I}=5$ (panel B). The color scale indicates the values of $p_{AB}$ which is computed by taking the mean value of the number of occurrences where the trajectory crosses from one differentiated state domain to another for 200 Gillespie simulations. The time window during which the number of transitions is counted starts at steady state (20-time units) and ends after 500-time units in order to broadly evaluate the number of transitions observed. Stability domains (black solid lines) are the same as in Fig 2. The black arrow indicates a reduction of $p_{AB}$ even if the system remains bistable as explained in the main text. Simulations were performed in the parametric space with a discretization step of 0.1 for *κ* and $F_{A}$.

To confirm this intuition, we computed the probability per unit of time $p_{AB}$ to observe a transition between states ***A*** and ***B***. This computation involves tracking the number of occurrences where the trajectory crosses from one differentiated state coarse-grained domain (defined by Eqs (11) and (12)) to another through the coarse-grained domain corresponding to the non-differentiated state (defined by Eqs (11) and (12)), within a specified time window Δt after which the system is considered stationary. Fig 14 shows how $p_{AB}$ depends on *κ* and $F_{A}$, for two different values of $F_{I}$. Fig 14A shows that transitions are much more frequent under conditions corresponding to bistability than when tristability is present, confirming our intuition.

Such graphs can also be used to assess the role of key parameters on transitions in the bistable domain. By comparing Figs 14A and 14B, we observe that these transitions become less frequent across the entire bistable domain as $F_{I}$ increases. This decrease is attributed to the increasing distance between the two differentiated states ***A*** and ***B*** (and therefore the distance between the differentiated states and the separatrix associated to the ***SC*** state). Indeed, the expanded separation between states poses a challenge for the transition process, as the trajectory must cover a greater distance before reaching the phase space separatrix (see Fig 3B and 3C). Consequently, more significant fluctuations are required to observe transitions between states when $F_{I}$ is large. Thus, transitions will be less frequent if the level of noise decreases in the system (for large values of Ω) and can completely disappear, as shown in S7 Fig. Furthermore, Fig 14A shows that for a relatively low $F_{I}$ value, the probability of observing a transition between ***A*** and ***B*** in the bistable domain depends on the proximity of the system to the tristable domain. Decreasing *κ* at constant $F_{A}$ (as illustrated by the black arrow in Fig 14A) results in a steep decrease of $p_{AB}$ as the tristability domain is approached. This decrease can, again, be associated with an increased distance between differentiated states ***A*** and ***B*** (or the distance between them and the separatrix) (see S2 Table for the effect of *κ* on steady-state values).

We conclude this section with a remark on the absence of A→C→B or B→C→A transitions in the tristable domains. Because a third state can be accessed (state ***C***), we need to assess whether transitions to and/or from this state can also occur. Indeed, state ***C*** must be stable enough to prevent transitions between ***A*** and ***B***, but should not be too attractive either, to prevent cells from being trapped in a non-differentiated state. To quantify how attractive this state is, we computed $\Delta n_{C}=n_{C}\left(t+\Delta t\right)-n_{C}\left(t\right)$ where $n_{C}$ is the fraction of cells in state ***C***. This quantity was calculated for times *t* that are larger than the time required for a cell to settle in one of the three available states for the first time. Positive values of $\Delta n_{C}$ indicate that state ***C*** is strongly attracting trajectories and gets populated over time, while negative values of this measure indicate that state ***C*** tends to rapidly give way to states ***A*** or ***B***. Moderately attracting ***C*** states are thus expected to correspond to $\Delta n_{C}\approx 0$.

**Fig 15:**
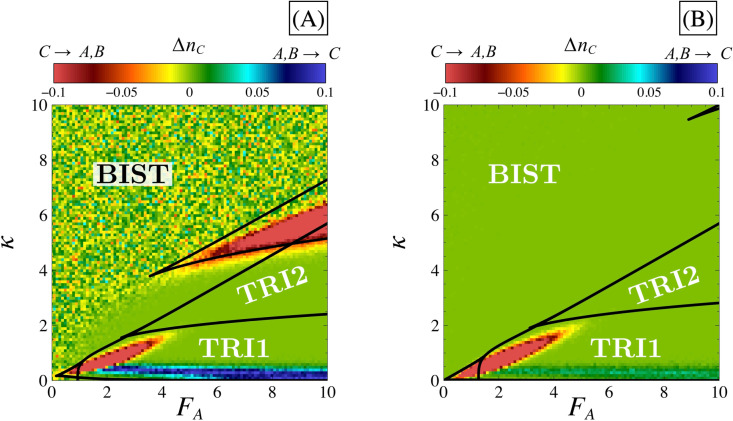
Transition between differentiated and non-differentiated states. Panels (A) and (B) display the mean value of $\Delta n_{C}=n_{C}\left(t_{s}+\Delta t\right)-n_{C}\left(t_{s}\right)$ from 2000 Gillespie simulation in the parametric space $F_{A},\kappa$ for different values of $F_{I}=2$ (panel A) and $F_{I}=5$ (panel B). The system is considered stationary after 20 times units ($t_{S}=20$) and each simulation stops at t=1000 (i.e., Δt=980). The extensivity parameter Ω=10. Red and orange/yellow zones ($\Delta n_{C}>0$) signify transitions from the non-differentiated state to the differentiated states, while blue and dark green zones ($\Delta n_{C}<0$) signify transitions from the differentiated states to the non-differentiated state. Light green zones indicate no observed transitions in the system. Stability domains (black solid lines) are the same as in Fig 2. Simulations were performed in the parametric space with a discretization step of 0.1 for *κ* and $F_{A}$.

In Fig 15, $\Delta n_{C}$ is depicted in the ($F_{A},\kappa$) parametric space for two different values of $F_{I}$ (and still for the symmetric systems). We focus here on the properties of the Tristable I case, where all three states are accessible. As shown in these diagrams, moderately attracting ***C*** states ($\Delta n_{C}\approx 0$) are found in a region corresponding to sufficiently large values of *κ* and $F_{A}$. This tendency can, again, be explained by the properties of phase space. In these parametric regions, the separatrices are relatively distant from the differentiated states, which are themselves also relatively distant from the non-differentiated state. Consequently, even in the presence of fluctuations, trajectories fail to cross one of the two separatrices, at least, during the time interval t+Δt in our simulations. Cells remain stuck in their primary choice and the differentiation process can be said to be robust against molecular noise. Combining these observations, we conclude that the non-differentiated state prevents transitions between the differentiated states. Indeed, for transitions such as A→C→B or B→C→A to occur, state ***C*** must be sufficiently attractive for the first phase of the transition (A→C or B→C) and poorly attractive for the second phase (C→B or C→A). This may suggest that a potential compromise between low and high attractivity might allow transitions from ***A*** to ***B*** or from ***B*** to ***A***. However, as explained above, a moderately attracting ***C*** state preclude this hypothesis, as within the parametric domain where $\Delta n_{C}\approx 0$, no transitions are observed.

However, as illustrated in S8 Fig which shows the equivalent of Fig 15 for higher values of Ω, the value of $\Delta n_{C}$ converges to 0 for all possible parametric configurations as the system size increases. This implies that transitions between the differentiated states and the non-differentiated state are intrinsically stochastic phenomena that manifest only in the presence of fluctuations.

#### Asymmetric case.

The previous subsection focused on transitions in a symmetric GRN, and one might wonder how asymmetries in the system affect transitions between states. In contrast to the symmetric case, the probabilities can be different in the two possible directions (***A*** to ***B*** versus ***B*** to ***A***). In this section, we briefly summarize our observations on that question.

In the bistable case, asymmetries promote transitions between states ***A*** and ***B*** when the distance between these differentiated states and the unstable non-differentiated state ***SC*** decreases. When this distance decreases in the presence of asymmetries, the separatrix moves closer to the differentiated state disfavoured by the asymmetries and further away from the state favoured by them. This shift increases the probability of transitions from the disfavoured state to the favoured one, while reducing the probability of transitions from the favoured state to the disfavoured one.

If we consider a scenario where trajectories initially lie in the state disadvantaged by the asymmetry, the proximity of the unstable state ***SC*** tends to deplete the population of this disadvantaged state, as was also observed in [48]. This phenomenon also occurs in the tristable case II, which includes an unstable state ***SC*** similar to the unstable state ***SC*** in the bistable case. Moreover, the depletion of one of the differentiated states is observed regardless of the nature of the asymmetry applied to the system.

In the tristable I case, two situations can be distinguished. When state ***C*** is strongly attractive, and as mentioned in the section on the effect of asymmetries on the phase portrait, the proximity between the differentiated states and their saddle points implies that they will move similarly under the effect of asymmetries. Thus, transitions from the differentiated states ***A*** or ***B*** to the non-differentiated state ***C*** are barely affected by asymmetries. However, the stable non-differentiated state ***C*** may be brought closer to the separatrices for certain types of asymmetries (S3 Fig and S2 Table). This leads to more frequent transitions from state ***C*** to the state favoured by the asymmetries. In the case of a less attractive ***C*** state, the positions of the saddle points and their differentiated steady states do not evolve in the same way under asymmetries (S3 Fig). It is now possible for certain asymmetries to bring the saddle points ***SA*** and ***SB*** closer to their differentiated states ***A*** and ***B***. Consequently, increased transitions from the differentiated state ***A*** or ***B*** to state ***C*** become possible. Furthermore, as in the attractive case, asymmetries that bring state ***C*** and the saddle points ***SA*** and ***SB*** closer favour transitions from state ***C*** to state ***A*** or ***B***, depending on which state is favoured.

In conclusion, whether the GRN is symmetric or not, it is crucial for a robust differentiation process that the steady states are not too close in phase space, which is linked to the attractivity of the non-differentiated state. Additionally, the inhibition and/or autoactivation parameters ($F_{I}$ or $F_{A}$, respectively) should be high enough to avoid transitions between states.

## Discussion

Understanding the robustness of the differentiation process in living systems requires a thorough analysis of the gene regulatory network (GRN) components that influence sensitivity to noise and asymmetries. Our study of a two-genes GRN reinforces existing research, highlighting the importance of autoactivations in strengthening network robustness during differentiation.

Crucially, we reveal that the stable non-differentiated state found in such GRNs plays a central role in shaping the system’s dynamics and ensuring robustness, particularly in the presence of asymmetries. This state reduces sensitivity to parametric perturbations, lowers the likelihood of transitions between states, and enhances overall system stability. Moreover, the establishment of a third basin of attraction linked to the non-differentiated state decreases sensitivity to initial perturbations or GRN configuration changes, influencing the system’s behaviour after its first decision. This contrasts sharply with systems where the non-differentiated state is unstable.

Without autoactivations or a stable non-differentiated state, the trajectory’s outcome becomes, on average, entirely predictable based on the GRN’s state (whether symmetric or asymmetric), even in the presence of noise. This predictability stems from the system starting in one of the two basins corresponding to a differentiated state, leaving no possibility for cells to occupy an intermediate non-differentiated basin at the beginning of their evolution, unlike in tristable systems.

Thus, tristability—and the autoactivations that enable it—are crucial for allowing the system to tolerate slight asymmetries in its initial state. This tolerance arises from the system’s ability to be moderately or strongly drawn towards the non-differentiated state, reducing the influence of initial asymmetries on its evolution.

Furthermore, we demonstrate that the impact of the non-differentiated state on the system properties is contingent upon its accessibility and attractivity, as well as the nature of the asymmetry that breaks the symmetry of the GRN. A highly attractive non-differentiated state, while rendering the differentiation process less effective, imparts significant robustness to cell proportions. In contrast, a non-attractive non-differentiated state, whether unstable or stable but inaccessible from low initial conditions, leads to a notable decrease in the robustness of cell proportions. Achieving a balance is therefore crucial, necessitating a stable non-differentiated state that is not excessively attractive to prevent inefficiencies in the differentiation mechanism, yet sufficiently attractive to safeguard its robustness. Interestingly, we have demonstrated that this balance is achieved when the system exhibits a ghost state. This state influences the direction of phase space trajectories by attracting them and slowing their dynamics in its vicinity. This deceleration creates an opportunity for noise to redirect the trajectories toward a differentiated state, highlighting the ghost state’s critical role in the process. In parallel, our exploration has underscored the indispensable consideration of intrinsic noise within the system. By considering the evolution of transcription factors as a stochastic phenomenon, we obtained a whole picture of the sensitivity of the system when the GRN is symmetric or asymmetric. The inclusion of stochastic noise appears as a key symmetry breaking factor allowing the emergence of balanced proportions where the system is asymmetric and/or strongly attracted by the non-differentiated state. Consequently, the presence of noise in the system introduces the possibility to leverage of the benefits of the attractivity of the non-differentiated state in terms of robustness while leaving aside the constraints that this state imposes on the differentiation phenomenon.

Following the initial decision step governed by low-time dynamics, tristability can enable cells, under specific parametric conditions and in the presence of noise, to differentiate if they have not already done so, or to de-differentiate if they have previously differentiated, regardless of whether the gene regulatory network (GRN) is symmetric or asymmetric. The only contribution of asymmetries lies in the possibility of favouring or disfavouring these types of transitions by changing the position of the different steady states in phase space. While having a stable non-differentiated state is clearly beneficial for the robustness of the differentiation process in terms of maintaining proportions, its role in the context of transitions is more nuanced. Indeed, depending on the biological system that the GRN is intended to model, it is essential to carefully select the parametric configuration, as the directionality of transitions—whether from differentiated to non-differentiated states or vice versa—will vary depending on these parametric conditions. Certain types of transitions may be desired or, conversely, prohibited. By exhaustively analysing the parametric space, our study makes it possible to precisely situate these different cases. For the parametric configurations for which no transitions are observed between the differentiated states (a large majority of the tristable I domain), our analysis demonstrated that a stable non-differentiated state can make the first cellular decision irreversible. Tristability therefore also makes it possible to strengthen the fate of the cell, thus making, for these parametric conditions, the differentiation process more robust.

The notable disparity observed between the bistable and tristable I regimes, across the different properties we examined, can be attributed to the profound transformation in the phase portrait. The distinctiveness arises from the altered characteristics when the non-differentiated state attains stability. Remarkably, the phase portrait becomes an informative canvas that reveals substantial insights without necessitating more sophisticated analytical or computational methods. Simply by exploring the phase portrait, a wealth of information can be extracted, offering a direct and intuitive glimpse into the system’s behaviour and dynamics. However, although investigating transitions within the phase portrait using statistical approaches is intuitive and provides useful information, several more detailed methods exist that can offer greater precision regarding these processes without requiring coarse graining of the phase space. Such approaches [42,43,46,47,50–52] involve analysing transitions that follow privileged trajectories and enable a deeper understanding of the pathways and rates of transitions between stable states, shedding light on how noise and system dynamics influence these trajectories. Such studies could be particularly useful for exploring how symmetry breaking and the attractivity of the non-differentiated state shape the system’s transition dynamics. This could pave the way for future work to build upon and refine the findings obtained from phase portrait analyses.

Our reparameterization of Huang’s model [3] enables us to isolate the role of symmetry-breaking in shaping system behaviour. By decoupling the parameters governing symmetry-breaking from those influencing the system’s stability, we provide a clearer understanding of how asymmetries drive the system’s dynamics and trajectories. This approach holds potential for broader applications in quantifying symmetry-breaking effects in other systems. Furthermore, an exhaustive exploration of the parametric space revealed the richness of Huang’s model, highlighting conditions under which tristability is achievable, the emergence of ghost states, and other intricate dynamical phenomena. For each parametric domain, our work addressed both static properties (e.g., population distributions and state stability) and dynamic properties (e.g., transitions between states) under perturbed and unperturbed scenarios. This comprehensive approach underscores the importance of integrating phase space analysis with statistical properties to fully capture the behaviour of GRNs under diverse conditions.

While this study focused on isolated cells and did not encompass the signalling processes that are undoubtedly present in biological systems, our analysis provides a profound insight into the fundamental regulatory mechanisms governing the differentiation process. Even though our model does not explicitly incorporate signalling, our asymmetry parameters can artificially reproduce the impact of signalling on different modules within our GRN. Several approaches currently exist to introduce signalling into systems, such as incorporating a parameter that artificially increases or decreases over time to reproduce the effect of the signalling on the symmetry-breaking in the network or a change in the system’s multistability [2,4,7,11,38,48,52,53] or by introducing new time-dependent variables whose dynamics could transform the overall system behaviour [10,13,20,33,43,47,48,54–57]. These different methods, although reproducing certain desired/observed properties of biological systems during the differentiation process, do not, to our knowledge, investigate the impact that signalling can have on the central network and all the possibilities that a simple regulatory network without signalling can offer. Therefore, this enhanced understanding of how a simple gene regulatory network responds to various asymmetries paves the way for future investigations considering alternative scenarios to integrate signalling pathways into the system that are more targeted to certain modules of the network, thus making the connection between models and biological systems more precise. Future investigations will aim to unravel the behaviours induced by signalling in a spatially extended system of coupled cells and how we can understand these new behaviours based on the core properties of the network that governs cell differentiation.

## Supporting information

S1 FigBifurcation diagram of $x_{s}-y_{s}$ as a function of the inhibition force for the bistable case.(PDF)

S2 FigBifurcation diagram of $x_{s}$ as a function of the dissociation constant ratio *κ.*(PDF)

S3 FigBifurcation diagrams of $x_{s}-y_{s}$ as a function of the asymmetry parameters.(PDF)

S4 FigRobustness diagrams for different values of $F_{I}$ and *Ω.*(PDF)

S5 FigProportions of A, B and C in the parametric space ( $F_{A}$ , *κ* ) for Ω=10 .(PDF)

S6 FigProportions of A, B and C in the parametric space ( $F_{A}$ , *κ* ) for Ω=100 .(PDF)

S7 FigTransition probabilities $p_{AB}$ for Ω=100 .(PDF)

S8 FigTransition between differentiated and non-differentiated states for Ω=100 .(PDF)

S1 Table Transition probabilities in the chemical master equation. (PDF)

S2 Table Approximative steady-state solutions for the bistable and the tristable cases. (PDF)
