# Supplementary figures and images for: Role of tristability in the robustness of the differentiation mechanism
Source: PLoS One. 2025 Mar 19;20(3):e0316666. doi: 10.1371/journal.pone.0316666 (PMC11922266; doi:10.1371/journal.pone.0316666)

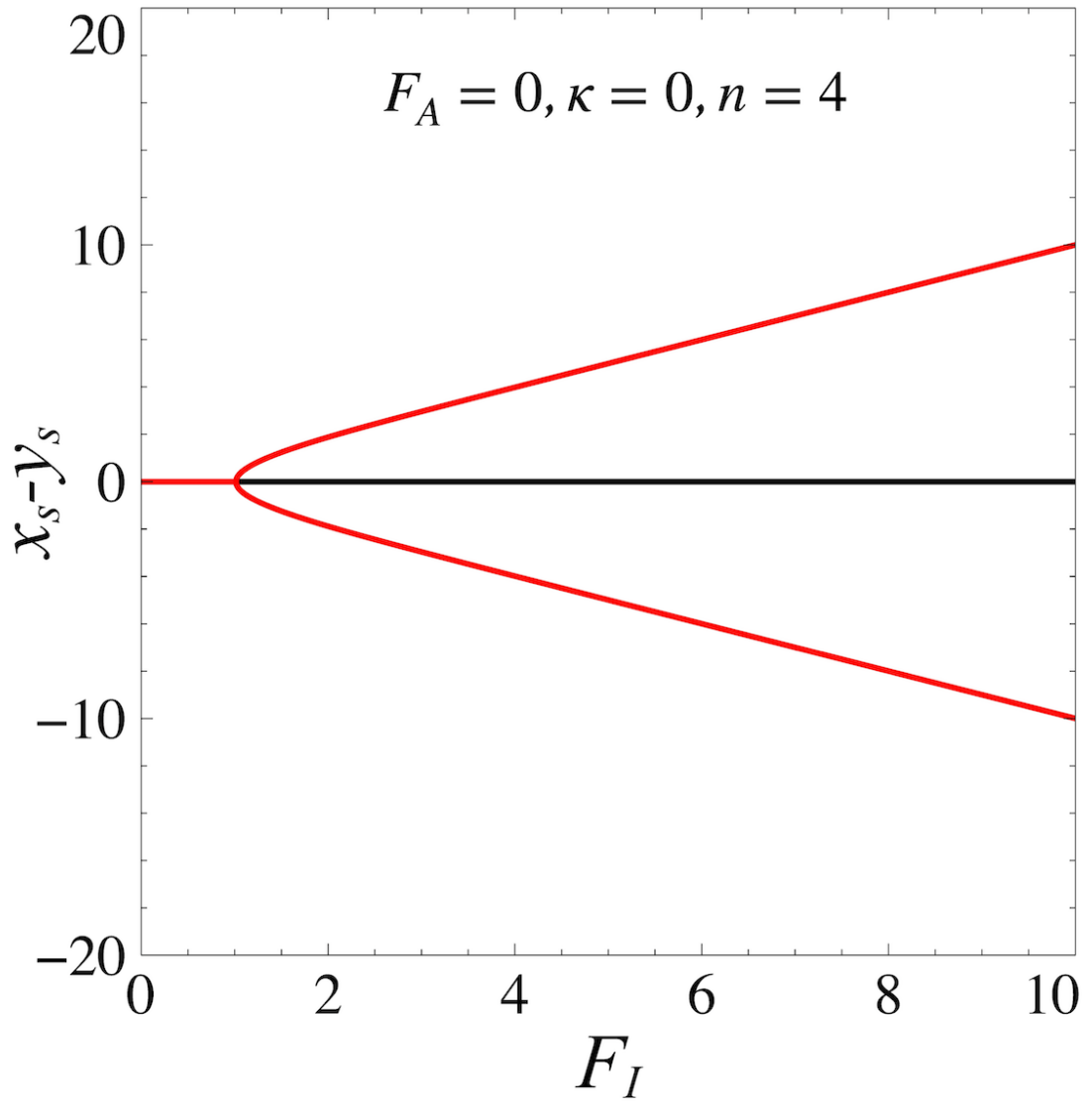

**S1 Fig. Bifurcation diagram of  $x_s - y_s$  as a function of the inhibition force for the bistable case.**

Supplement: S1 Fig — (PDF) [file pone.0316666.s001.pdf]

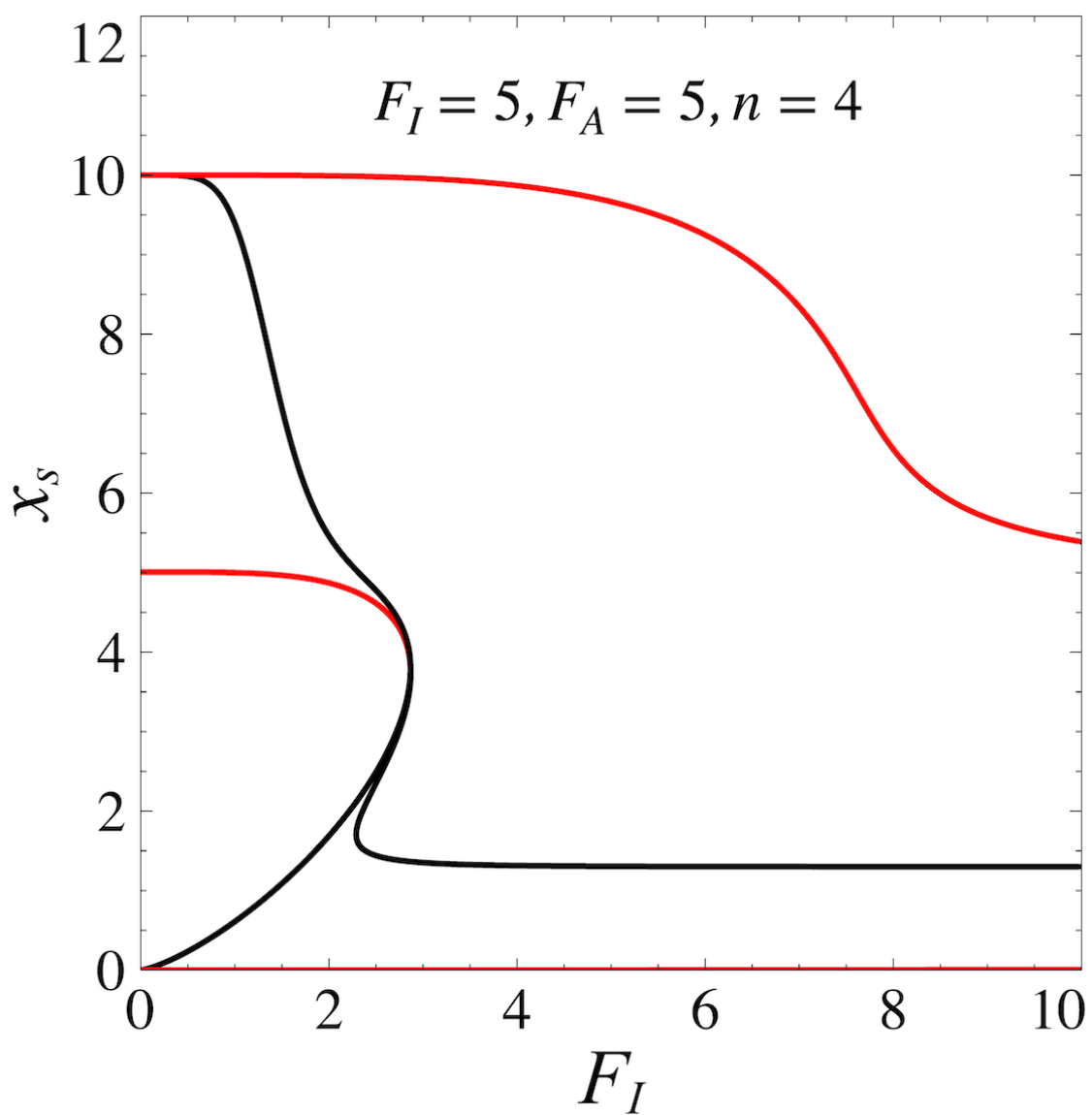

S2 Fig. Bifurcation diagram of  $x_s$  as a function of the dissociation constant ratio  $\kappa$

Supplement: S2 Fig — (PDF) [file pone.0316666.s002.pdf]

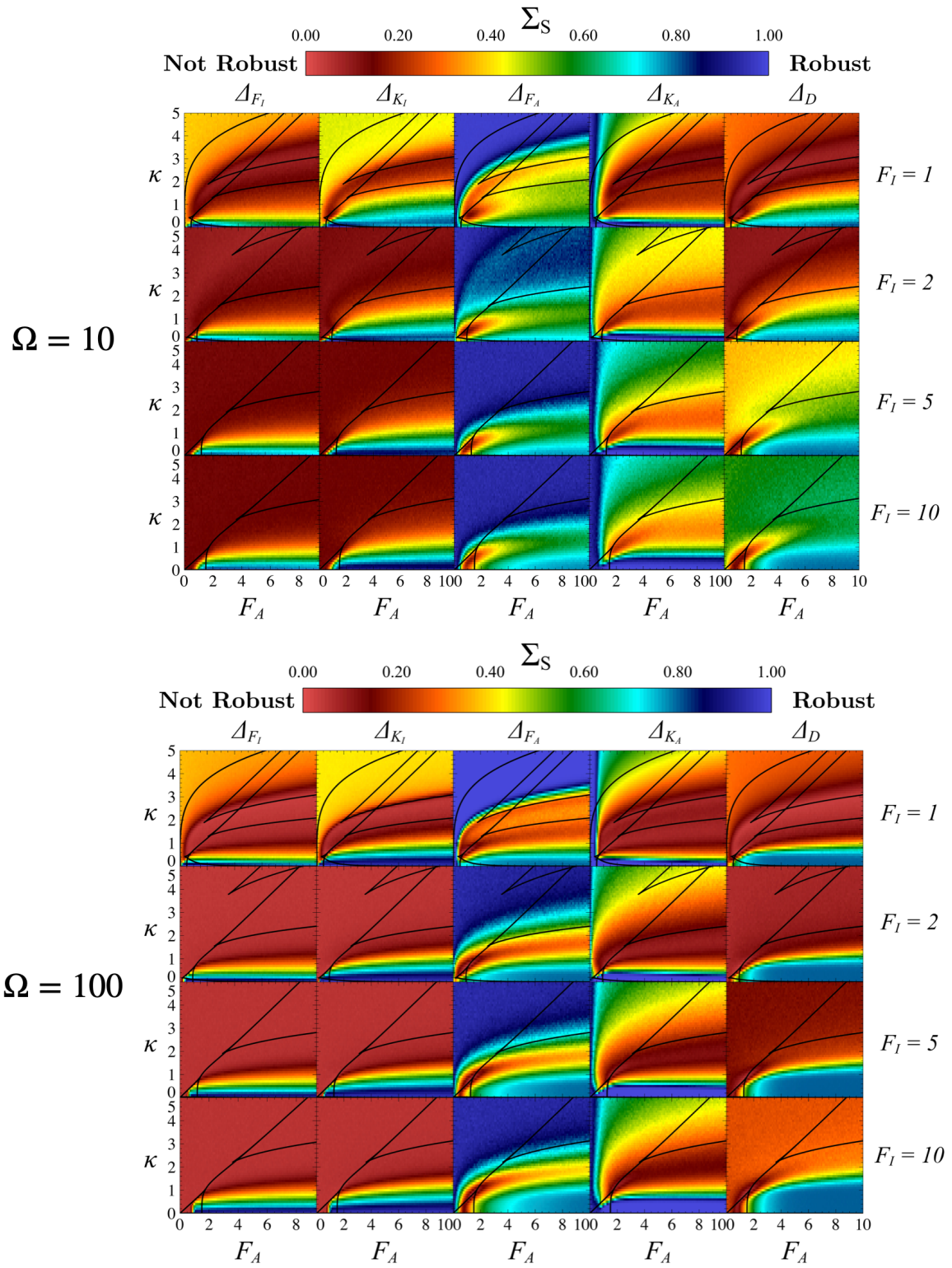

S4 Fig. Robustness diagrams for different values of  $F_I$  and  $\Omega$

Supplement: S4 Fig — (PDF) [file pone.0316666.s004.pdf]

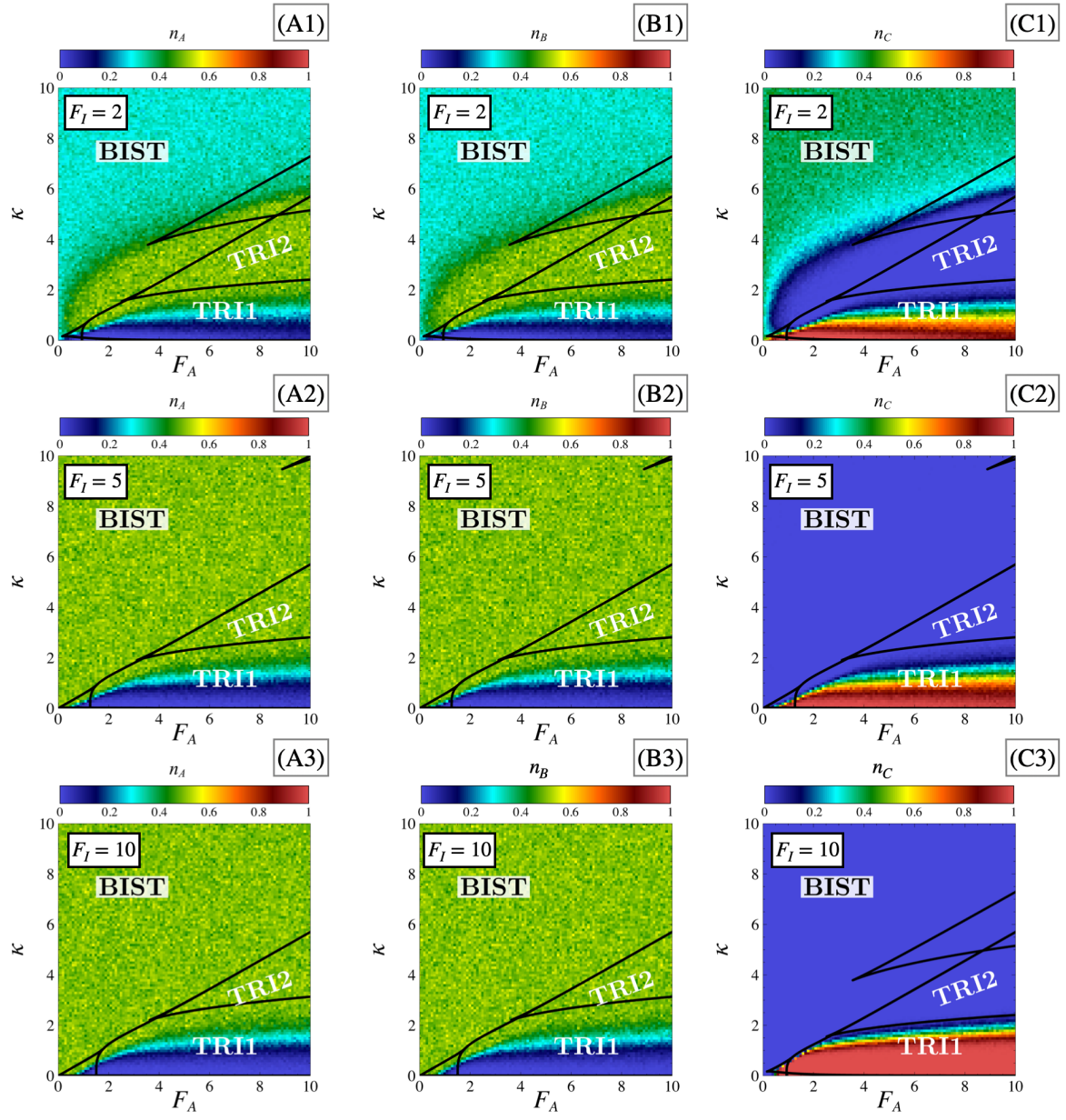

S5 Fig. Proportions of A, B and C in the parametric space ( $F_A, \kappa$ ) for  $\Omega = 10$

Supplement: S5 Fig — (PDF) [file pone.0316666.s005.pdf]

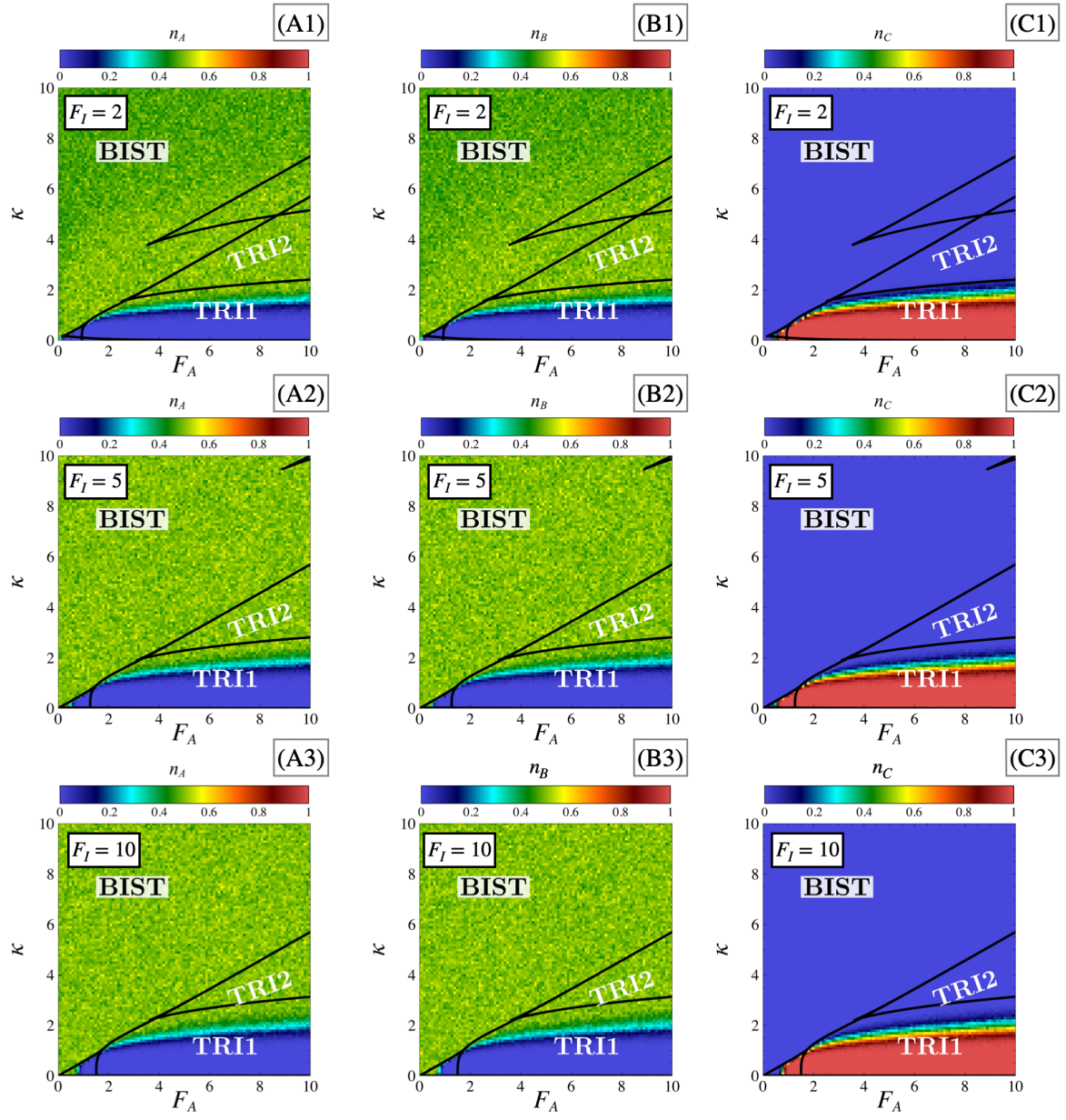

S6 Fig. Proportions of A, B and C in the parametric space ( $F_A, \kappa$ ) for  $\Omega = 100$

Supplement: S6 Fig — (PDF) [file pone.0316666.s006.pdf]

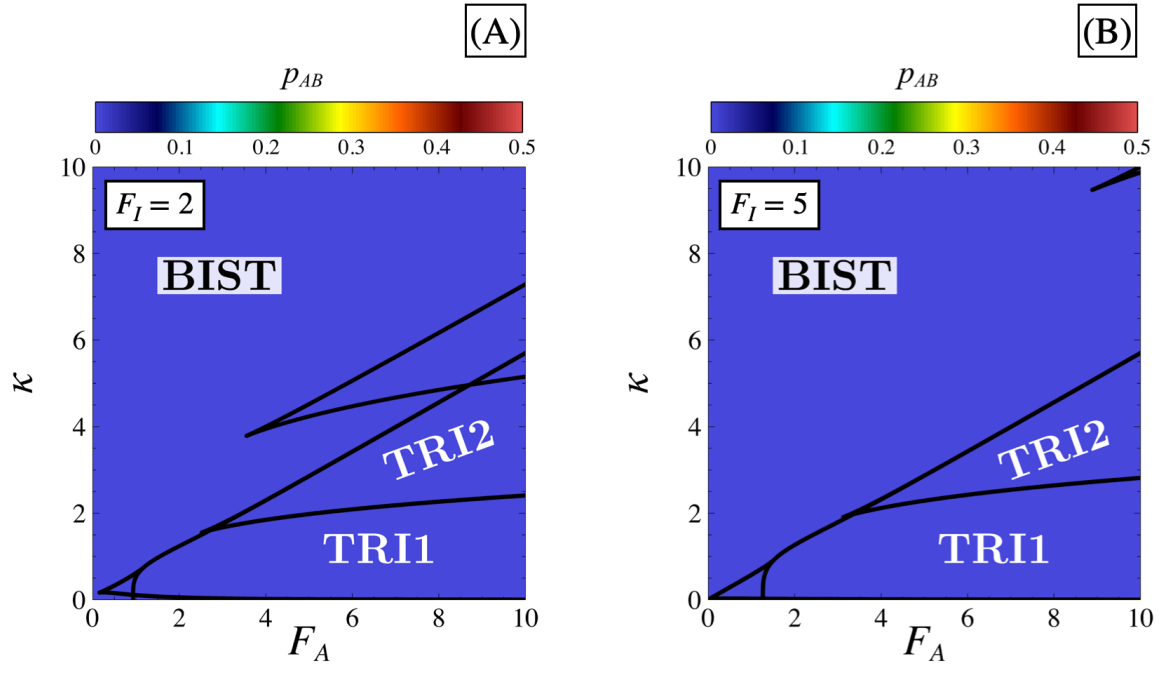

S7 Fig. Transition probabilities  $p_{AB}$  for  $\Omega = 100$ .

Supplement: S7 Fig — (PDF) [file pone.0316666.s007.pdf]

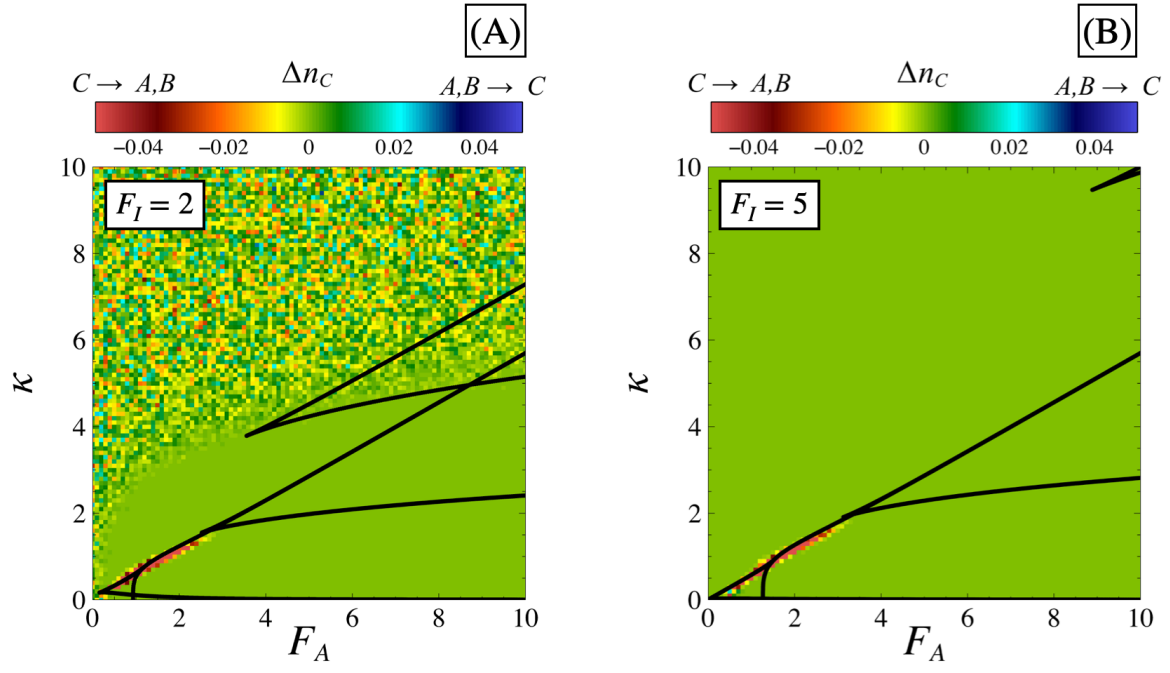

**S8 Fig. Transition between differentiated and non-differentiated states for  $\Omega = 100$**

Supplement: S8 Fig — (PDF) [file pone.0316666.s008.pdf]
