# Supplementary material for: Role of tristability in the robustness of the differentiation mechanism
Source: PLoS One. 2025 Mar 19;20(3):e0316666. doi: 10.1371/journal.pone.0316666 (PMC11922266; doi:10.1371/journal.pone.0316666)
Supplement: S3 Fig — (PDF) [file pone.0316666.s003.pdf]

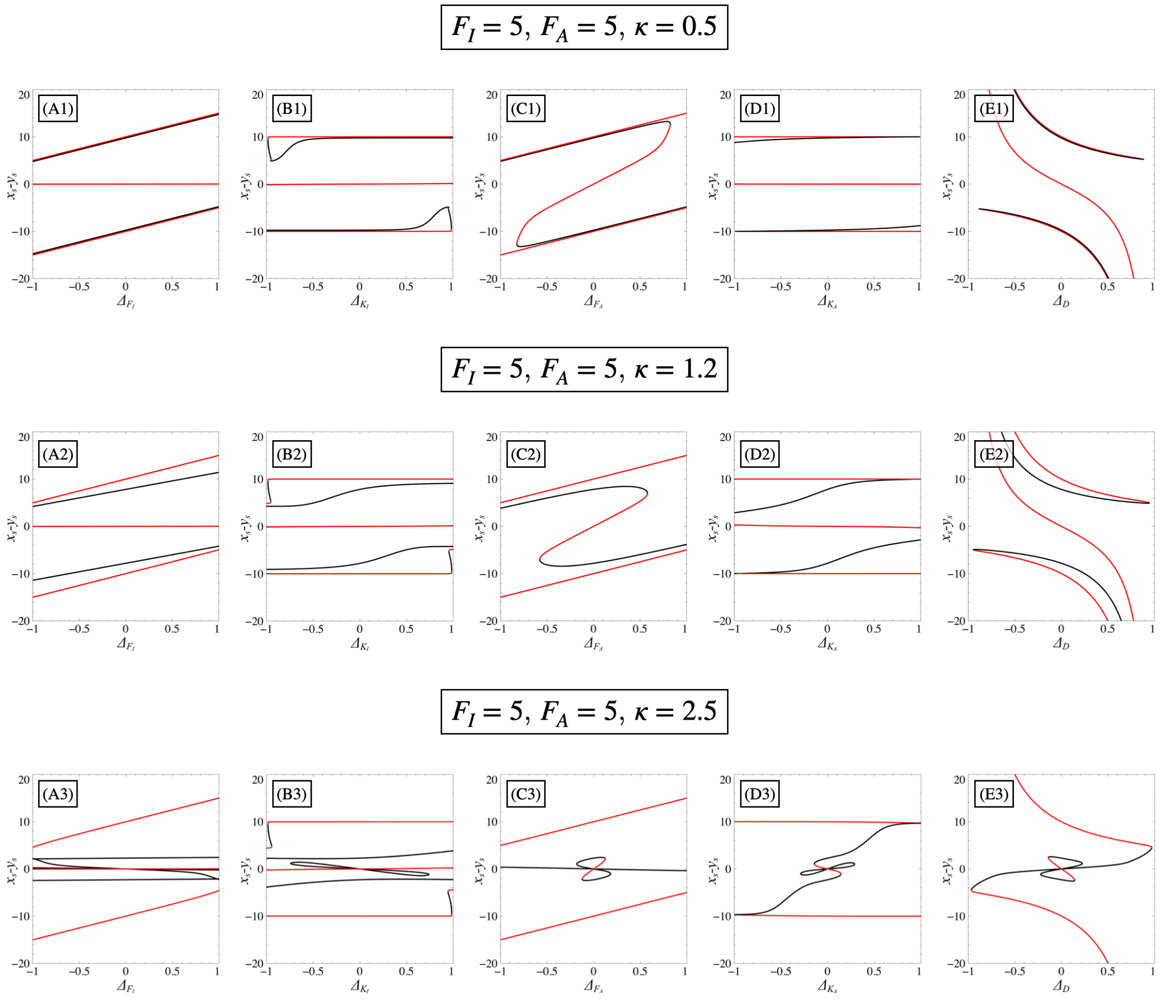

S3 Fig. Bifurcation diagrams of $x_s - y_s$ as a function of the asymmetry parameters.
