# Supplementary material for: Role of tristability in the robustness of the differentiation mechanism
Source: PLoS One. 2025 Mar 19;20(3):e0316666. doi: 10.1371/journal.pone.0316666 (PMC11922266; doi:10.1371/journal.pone.0316666)
Supplement: S1 Table — (PDF) [file pone.0316666.s009.pdf]

| | **Effect** | **Transition probabilities per unit of time $W_\rho$** | **Mathematical Expression** |
|---|---|---|---|
| Process $\rho_1$ (Inhibition) | $X \to X+1$ | $W_1(X,Y\,|\,X-1,Y)$ | $\Omega F_I(1+\Delta_{FI})\dfrac{(1+\Delta_{KI})^n}{(1+\Delta_{KI})^n+(Y\Omega^{-1})^n}$ |
| Process $\rho_2$ (Inhibition) | $Y \to Y+1$ | $W_2(X,Y\,|\,X,Y-1)$ | $\Omega F_I(1-\Delta_{FI})\dfrac{(1-\Delta_{KI})^n}{(1-\Delta_{KI})^n+(X\Omega^{-1})^n}$ |
| Process $\rho_3$ (Activation) | $X \to X+1$ | $W_3(X,Y\,|\,X-1,Y)$ | $\Omega F_A(1+\Delta_{FA})\dfrac{\left((X-1)\Omega^{-1}\right)^n}{\kappa^n(1+\Delta_{KA})^n+\left((X-1)\Omega^{-1}\right)^n}$ |
| Process $\rho_4$ (Activation) | $Y \to Y+1$ | $W_4(X,Y\,|\,X,Y-1)$ | $\Omega F_A(1-\Delta_{FA})\dfrac{\left((Y-1)\Omega^{-1}\right)^n}{\kappa^n(1-\Delta_{KA})^n+\left((Y-1)\Omega^{-1}\right)^n}$ |
| Process $\rho_5$ (Degradation) | $X \to X-1$ | $W_5(X,Y\,|\,X+1,Y)$ | $(1+\Delta_D)(X+1)$ |
| Process $\rho_6$ (Degradation) | $Y \to Y-1$ | $W_6(X,Y\,|\,X,Y+1)$ | $(1-\Delta_D)(Y+1)$ |

**S1 Table. Transition probabilities in the chemical master equation.**
