# Supplementary material for: Role of tristability in the robustness of the differentiation mechanism
Source: PLoS One. 2025 Mar 19;20(3):e0316666. doi: 10.1371/journal.pone.0316666 (PMC11922266; doi:10.1371/journal.pone.0316666)
Supplement: S2 Table — (PDF) [file pone.0316666.s010.pdf]

Regime	State	x_s value	y_s value	Asymmetry dependence
Bistable Regime $(F_A = 0)$ or $(F_A \neq 0)$ $(\kappa \ggg 1)$	**A State**	$F_I \frac{\delta_{F_I}}{\delta_D}$	0	Δ_{F_I}, Δ_D
	**B State**	0	$F_I \frac{\bar{\delta}_{F_I}}{\bar{\delta}_D}$	Δ_{F_I}, Δ_D
	**C State (Saddle)**	$\sqrt[n+1]{F_I \frac{\delta_{F_I}\delta_{K_I}^n}{\delta_D}}$	$\sqrt[n+1]{F_I \frac{\bar{\delta}_{F_I}\bar{\delta}_{K_I}^n}{\bar{\delta}_D}}$	$\Delta_{F_I}, \Delta_{K_I}, \Delta_D$
Tristable 1 Regime $(F_A \neq 0)$ $(\kappa < 1)$	**A State**	$F_I \frac{\delta_{F_I}}{\delta_D} + F_A \frac{\delta_{F_A}}{\delta_D}$	0	$\Delta_{F_I}, \Delta_{F_A}, \Delta_D$
	**B State**	0	$F_I \frac{\bar{\delta}_{F_I}}{\bar{\delta}_D} + F_A \frac{\bar{\delta}_{F_A}}{\bar{\delta}_D}$	$\Delta_{F_I}, \Delta_{F_A}, \Delta_D$
	**C State (Stable)**	$F_A \frac{\delta_{F_A}}{\delta_D}$	$F_A \frac{\bar{\delta}_{F_A}}{\bar{\delta}_D}$	Δ_{F_A}, Δ_D
Intermediate Regime $(F_A \neq 0)$ $(\kappa > 1)$	**A State**	$F_I \frac{\delta_{F_I}}{\delta_D} + F_A \frac{\delta_{F_A}}{\delta_D}$	0	$\Delta_{F_I}, \Delta_{F_A}, \Delta_D$
	**B State**	0	$F_I \frac{\bar{\delta}_{F_I}}{\bar{\delta}_D} + F_A \frac{\bar{\delta}_{F_A}}{\bar{\delta}_D}$	$\Delta_{F_I}, \Delta_{F_A}, \Delta_D$
	**C State (Stable)**	$F_A \frac{\delta_{F_A}}{\delta_D} \frac{x_s{}^n}{\delta_{K_A}^n \kappa^n + x_s^n}$	$F_A \frac{\bar{\delta}_{F_A}}{\bar{\delta}_D} \frac{y_s{}^n}{\bar{\delta}_{K_A}^n \kappa^n + y_s^n}$	$\Delta_{F_A}, \Delta_{K_A}, \Delta_D$
Tristable 2 Regime $(F_A \neq 0)$ $(\kappa \gg 1)$	**A State**	$F_I \frac{\delta_{F_I}}{\delta_D} + F_A \frac{\delta_{F_A}}{\delta_D} \frac{x_s{}^n}{\delta_{K_A}^n \kappa^n + x_s^n}$	0	$\Delta_{F_I}, \Delta_{F_A}, \Delta_{K_A}, \Delta_D$
	**B State**	0	$F_I \frac{\bar{\delta}_{F_I}}{\bar{\delta}_D} + F_A \frac{\bar{\delta}_{F_A}}{\bar{\delta}_D} \frac{y_s{}^n}{\bar{\delta}_{K_A}^n \kappa^n + y_s^n}$	$\Delta_{F_I}, \Delta_{F_A}, \Delta_{K_A}, \Delta_D$
	**C State (Stable)**	$\sqrt[1-n]{F_A \kappa^n \frac{\delta_{F_A}}{\delta_D \delta_{K_A}^n}}$	$\sqrt[1-n]{F_A \kappa^n \frac{\bar{\delta}_{F_A}}{\bar{\delta}_D \bar{\delta}_{K_A}^n}}$	$\Delta_{F_A}, \Delta_{K_A}, \Delta_D$
	**C State (Saddle)**	$\sqrt[n+1]{F_I \frac{\delta_{F_I}\delta_{K_I}^n}{\delta_D}}$	$\sqrt[n+1]{F_I \frac{\bar{\delta}_{F_I}\bar{\delta}_{K_I}^n}{\bar{\delta}_D}}$	$\Delta_{F_I}, \Delta_{K_I}, \Delta_D$
Bistable Regime $(F_A \neq 0)$ $(\kappa \gg 1)$	**A State**	$F_I \frac{\delta_{F_I}}{\delta_D} + F_A \frac{\delta_{F_A}}{\delta_D} \frac{x_s{}^n}{\delta_{K_A}^n \kappa^n}$	0	$\Delta_{F_I}, \Delta_{F_A}, \Delta_{K_A}, \Delta_D$
	**B State**	0	$F_I \frac{\bar{\delta}_{F_I}}{\bar{\delta}_D} + F_A \frac{\bar{\delta}_{F_A}}{\bar{\delta}_D} \frac{y_s{}^n}{\bar{\delta}_{K_A}^n \kappa^n}$	$\Delta_{F_I}, \Delta_{F_A}, \Delta_{K_A}, \Delta_D$
	**C State (Saddle)**	$\sqrt[n+1]{F_I \frac{\delta_{F_I}\delta_{K_I}^n}{\delta_D}}$	$\sqrt[n+1]{F_I \frac{\bar{\delta}_{F_I}\bar{\delta}_{K_I}^n}{\bar{\delta}_D}}$	$\Delta_{F_I}, \Delta_{K_I}, \Delta_D$

S2 Table. Approximative steady-state solutions for the bistable and the tristable cases.

In this table, all the $\delta = (1 + \Delta)$ and all the $\bar{\delta} = (1 - \Delta)$
